# Optimized Milling Approaches for Scalable Production of Ritonavir Nanocrystals: from Process Design to Bioperformance Evaluation

**DOI:** 10.1021/acsomega.5c09971

**Published:** 2026-01-27

**Authors:** Marcelo Henrique da Cunha Chaves, Francisco Alexandrino-Júnior, Michelle Alvares Sarcinelli, Natalia Cristina Gomes-da-Silva, Ralph Santos-Oliveira, Fabio Coelho Amendoeira, Helvécio Vinícius Antunes Rocha

**Affiliations:** † Laboratory of Micro and Nanotechnology, 37903 Oswaldo Cruz Foundation/Fiocruz, Rio de Janeiro 21040-900, Brazil; ‡ Postgraduate Program in Health Surveillance, National Institute for Quality Control in Health, Oswaldo Cruz Foundation/Fiocruz, Rio de Janeiro 21040-900, Brazil; § Brazilian Nuclear Energy Commission, Nuclear Engineering Institute, Laboratory of Nanoradiopharmacy and Synthesis of New Radiopharmaceuticals, Rio de Janeiro-RJ 21941906, Brazil; ∥ State University of Rio de Janeiro, Laboratory of Nanoradiopharmacy and Strategic Biomaterials, Rio de Janeiro-RJ 220000, Brazil

## Abstract

The development of nanoformulations aims to overcome the biopharmaceutical limitations associated with conventional drug delivery. Reducing the particle size to the nanometric scale enhances drug solubility, dissolution rate, and bioavailability. In this study, the development and quality control of ritonavir nanocrystals are described by using applied experimental milling approaches. Ritonavir nanosuspensions were initially prepared at a small scale using an Ultra-Turrax Tube Drive, in which 200 and 500 μm beads were identified as the most efficient for particle size reduction. The process was then successfully scaled up by using a bead mill, achieving particle sizes of approximately 300 nm within 30 min, followed by spray-drying. Solid-state characterization by XRD, TGA, DSC, and hot-stage microscopy confirmed that the nanocrystals retained Form II ritonavir throughout processing. The resulting nanocrystals were physically stable and exhibited a marked improvement in dissolution, particularly in a discriminative dissolution medium (0.04 M POE10LE). Process optimization was achieved by balancing bead sizes and steric stabilizers, such as PVP K30 and HPMC. Biodistribution studies using [^99^mTc] radiolabeling (labeling efficiency >90%) showed uptake in the liver, kidneys, and intestines, with notable differences in the cardiac distribution between nanocrystals and nanosuspensions. Pharmacokinetic analysis indicated similar overall distribution profiles, with a transient 4 h peak in blood levels for the nanocrystals. Biochemical analyses suggested formulation-dependent hepatic stress, reflected by increased GGT for the nanosuspension, and alterations in carbohydrate metabolism, including elevated glucose and amylase levels, for the nanocrystal formulation. Overall, ritonavir nanocrystals significantly improved dissolution, and the optimized milling and spray-drying approach represents a robust and scalable strategy to enhance the performance of Class II or IV drugs.

## Graphical abstract

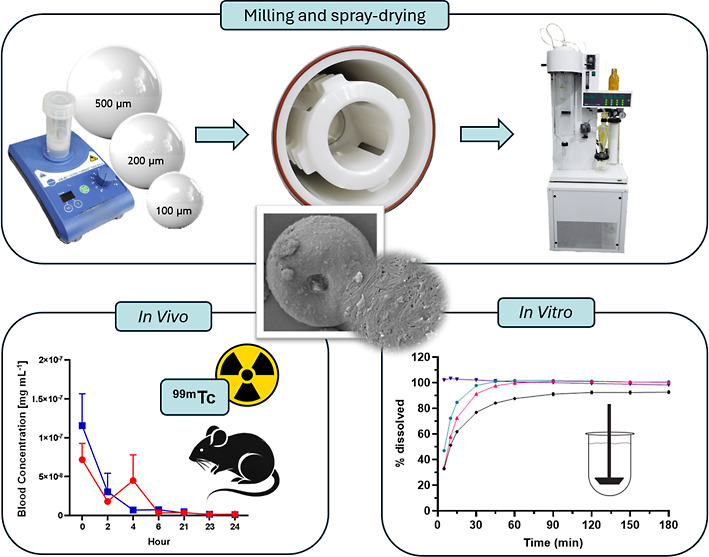

## 1 Introduction

Ritonavir (RTV) is a potent and effective HIV protease inhibitor, developed by Abbott Laboratories in 1992 and commercialized in 1996. Since its introduction, RTV has been widely used, often in combination with other antiretroviral drugs because of its CYP3A inhibition property, enhancing the pharmacokinetics of coadministered drug.^1^ This pharmacokinetic booster effect has extended the applications of RTV beyond HIV treatment, as demonstrated by its combination with nirmatrelvir in Paxlovid,^2^ which received emergency use authorization from the FDA in 2021 for the treatment of infections caused by severe acute respiratory syndrome coronavirus 2 (SARS-CoV-2).^3^

Despite its clinical success, formulating RTV as an effective dosage form presents significant challenges due to its physicochemical properties. Mainly because it displays polymorphism and is a class-IV drug in the biopharmaceutics classification system evidencing its low water solubility and unsatisfactory permeability.^4–6^ In fact, these characteristics have impacted on its manufacturability, as highlighted by the market crisis underwent by Abbot in early 1998 to when a previously unknown RTV polymorph (Form II) emerged in the semisolid capsule, drastically reducing its solubility and leading to unexpected dissolution test failures.^7^

To overcome the limitations associated with the poor drug bioavailability, innumerous industrial-scale formulation strategies have been implemented, including the production of amorphous solid dispersions (ASDs) and lipid-based drug delivery systems. However, these approaches face challenges such as production costs, stability issues inherent to amorphous systems formulations,^8^ and the substantial investment required for specialized manufacturing equipment. Additionally, the high cost of lipid excipients further limits the implementation of lipid-based formulations.^9^

In face of this scenario, an alternative involves the production of micro and nanocrystals,^10^ which can be achieved through both bottom-up and top-down approaches.^11^ In the former, nanostructures are produced from active pharmaceutical ingredient (API) molecules. This approach is less commonly used due to difficulties in scaling up and the need for organic solvents.^12,13^ In the top-down approach, larger API particles are fragmented into smaller particles until they reach the micro or nanoscale.^11^ To stabilize the system, stabilizing agents are employed to reduce the surface tension and prevent aggregation. The primary advantages of this latter methodology are its relative ease of scaling and reproducibility.^11,14,15^

From an economic standpoint, nanocrystals have emerged in the pharmaceutical market due to their ability to facilitate the use of drugs with low water solubility, thereby improving the bioavailability of their respective medications. Furthermore, unlike other nanoparticulate systems, nanocrystals are composed almost entirely of the API, with only small amounts of stabilizers in their formulations, which allow the formation of nanosuspensions in aqueous media.^16^

The immense potential of nanocrystals to address pharmaceutical limitations, such as poor solubility, slow dissolution, and reduced bioavailability, lies in the significant physicochemical properties they acquire at the nanoscale.^16^ In general, these improvements result from a substantial increase in the saturation concentration and dissolution rate, as well as an enhanced bioadhesion potential observed on the surface of cell membranes.^17^ These new properties are attributed to the nanoscale size and are related to the pronounced reduction of the diffusion layer surrounding the particles, as well as the significant increase in the surface area and curvature of the nanocrystals.^18^

This study aimed to reduce the particle size of RTV to the nanoscale, utilizing milling techniques (top-down), where the issues of RTV’s low oral bioavailability and poor water solubility can be addressed to provide a formulation with proven efficacy and safety. Additionally, it could serve as a model for other drugs, even those in other therapeutic categories, that belong to Class II or IV according to the BCS.^4,15,19^

As an innovative aspect, this work sought to delve deeper into the milling process itself, using different stabilizers, various bead sizes (and combinations), spray-drying the nanosuspensions, and characterizing the resulting particles. Ultimately, besides providing an optimized milling model, the study aimed to conduct trials with various batch sizes to enable scaling up of the milling techniques and generate alternatives for utilizing small quantities of drugs, thus allowing future milling tests to be performed with products derived from chemical synthesis laboratories in their early stages.

Therefore, this work addresses key aspects of pharmaceutical nanotechnology by proposing, for the first time, a scalable process for producing ritonavir nanocrystals, evaluating the effects of bead size and combinations and applying, for the first time, spray drying to ritonavir nanosuspensions. Additionally, by uniquely integrating radiolabeling techniques to assess ritonavir nanocrystal bioperformance, this study provides valuable insights into the in vivo behavior of these nanocrystals, contributing significantly to the development of more effective drug delivery systems.

## 2 Materials and Methods

### 2.1 Material

Sodium starch glycolate (SSG), Roquette; benzalkonium chloride (BAC), Merck; croscarmellose sodium (CCS), Alpha Hi-tech; hydroxypropyl cellulose (HPC LF), Ashland; hydroxypropyl cellulose (HPC SSL), Nisso; hydroxypropyl methylcellulose (HPMC 603), Shin Etsu; polyoxyethylene 10 lauryl ether (POE10LE), Sigma-Aldrich; sodium lauryl sulfate (SLS), VETEC; polyvinylpyrrolidone K30 (PVP K30), Xiao Fine Chemicals; Polysorbate 80 (TWEEN 80), Croda; Poloxamer 188 (POL188), Merck; Poloxamer 407 (POL407), Merck; and Ritonavir, Shanghai Desano Chemical Pharmaceutical.

### 2.2 Selection of Charge and Steric Stabilizers

The formulation process initiated with the identification and selection of appropriate stabilizers (charge and steric), guided by a comprehensive literature review and laboratory availability.^20^ This systematic approach led to the selection of the following stabilizers: BAC, CCS, HPC LF, HPC SSL, HPMC 603, PVP K30, POL188, POL407, SLS, SSG, and TWEEN 80.

The preliminary experiments revealed that TWEEN 80 and BAC exhibited excessively high absorbance at the UV wavelength of interest for RTV (0,607 and 0,276 nm, respectively, at 0,2% m/v), prompting its immediate exclusion from further consideration. Considering the higher costs and the fact that HPLC is a more time-consuming method compared with UV spectrophotometry, the latter was prioritized. Consequently, excipients that could cause interference were excluded from the experimental panel.

To enhance the intelligibility and facilitate traceability of the implemented methodological workflow, including successive processing stages and sample characterization, a schematic overview is presented in Figure 1.

**Figure 1:**
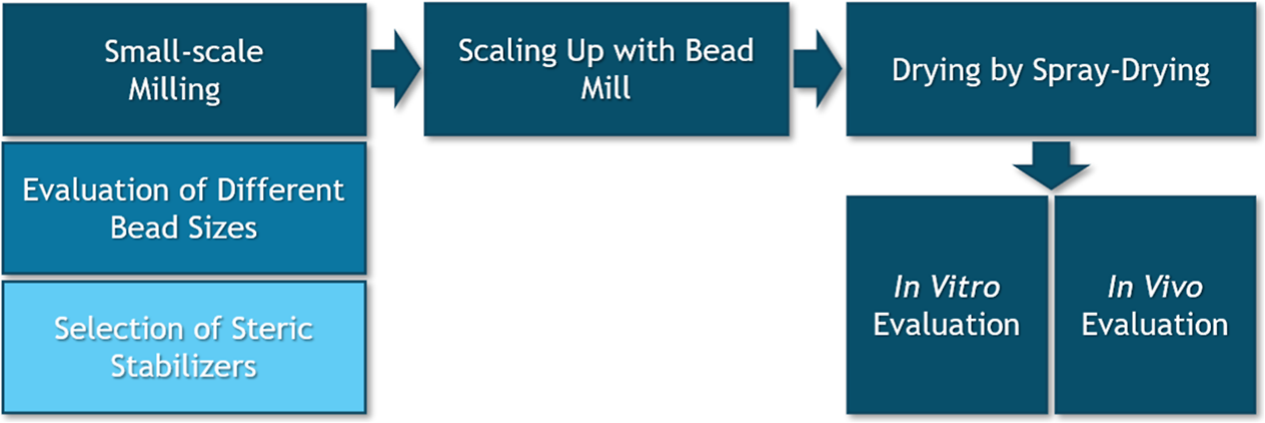
Schematic representation of the methodological workflow, illustrating the sequential processing stages and sample characterization.

### 2.3 Sample Processing Using a Ultra-Turrax Tube Drive Disperser

The primary objective of the initial investigations was to assess, in small-volume batches (10 mL), the effect of different stabilizers, bead sizes, and auxiliary procedures to identify optimal presuspension formulation and processes for RTV milling. This optimization enabled subsequent batch scale-up procedures.

All processing (Sections 2.3.1 and 2.3.2) used presuspensions containing 10% RTV (w/v), 2% charge stabilizer (w/w), and 5% steric stabilizer (w/w).^20^

#### 2.3.1 Evaluation of Bead Size Influence on Nanosuspension Milling

This phase focuses on investigating the influence of bead size dimensions on the nanosuspension milling efficiency. To reach this goal, three bead diameters were evaluated: 100, 200, and 500 μm.

To isolate the effects of bead size, a standardized mixture of stabilizers was used, consisting of SLS as the charge stabilizer and PVP-K30 as the steric stabilizer.

The experimental procedure involved completely dissolving stabilizers in 10 mL of purified water using magnetic stirring followed by the gradual RTV dispersion. Subsequently, the Ultra-Turrax Tube Drive (IKA) disperser was assembled, and 5 mL of yttrium-stabilized zirconium oxide beads (Netzsch) were transferred to the loading chamber, followed by the addition of the RTV dispersion containing stabilizers. The presuspension underwent processing through multiple 29 min milling cycles at maximum equipment velocity (6000 rpm) until particle size stabilization was observed.^20^

Particle size characterization was performed after each cycle using Mastersizer 3000 (Malvern) laser diffraction analysis and Zetasizer Nano ZS90 (Malvern) when the D90 values approached 1 μm.

This study also investigated the implementation of a premilling step, using an Ultra-Turrax T 25 disperser (IKA) to facilitate the initial milling stages. Furthermore, sequential milling processes varying the bead size were conducted, progressing from larger to smaller beads.

#### 2.3.2 Selection of Steric Stabilizers

To evaluate the effectiveness of different steric stabilizers for RTV nanocrystal processing, suspensions of the steric stabilizers, as presented in Section 2.2, were tested, keeping the same proportions as outlined in Section 2.3, i.e., RTV 10% w/v, charge stabilizer SLS 2%w/w, and steric stabilizer 5%w/w.^20^

### 2.4 Scaling-Up Process Using Bead Mill

Following the identification of promising presuspension formulations, the process was scaled up to 500 mL batches using a bead mill (Delta Vita 300, Netzsch) maintaining the established rations.^20^ This batch size was selected to balance the high cost of the API with the minimum volume required for an efficient subsequent drying process.

The scale-up protocol was initiated with the full dissolution of the stabilizers in 500 mL of purified water using a magnetic stirrer, followed by gradual RTV dispersion. To enhance processability for the milling stage, the resulting suspension underwent predispersion using an Ultra-Turrax T25 disperser (3 min at 11,000 rpm) to achieve homogeneous substrate consistency for subsequent processing. Throughout this preparation phase, the presuspension was kept under agitation.

The Delta Vita 300 milling chamber (maximum capacity of 300 mL) was loaded with 200 mL of beads. Following the chamber sealing, the system operated in a closed-circuit configuration, maintaining the mixture under continuous agitation and refrigeration, initially set to −6 °C. The peristaltic pump was set to 50 rpm, and the initial rotor speed was 1000 rpm. Nevertheless, subsequent experiments investigated the effect of varying rotor speeds to optimize the milling process.

Throughout the scale-up process, samples were collected at 30 min intervals to monitor trends in the stabilization of the nanocrystal size reduction. Comprehensive particle size and stability analyses were conducted across all processing trials (Sections 2.3 and 2.4). These analyses included the determination of D10, D50, and D90 values via laser diffraction and the calculation of the polydispersity index (span). Additionally, hydrodynamic particle size (*z*-average) and polydispersity index (PDI) were assessed via dynamic light scattering, alongside zeta potential (mV) measurements obtained through electrophoretic mobility.

### 2.5 Drying Nanosuspensions by Spray-Drying

The scaled-up nanosuspensions underwent drying using a Mini Spray Dryer B-290 (Buchi). The nanosuspensions were maintained under continuous agitation, while being introduced into the system via a peristaltic pump operating at 20% capacity (∼4 mL/min). The drying parameters were configured with an inlet temperature of 140 °C (outlet of 88 ± 2 °C), while the vacuum pump was operating at full capacity (aspiration of 100%). Upon process completion, the dried nanocrystals were recovered from the collection vessel and immediately transferred to hermetically sealed containers, which were subsequently stored in a vacuum desiccator containing silica gel as a desiccant to prevent moisture absorption.

### 2.6 Sample Characterization

#### 2.6.1 Particle Size Distribution Analysis via Laser Diffraction

The particle size distributions were determined by using laser diffraction (LD) analysis with a MasterSizer 3000 particle size analyzer (Malvern Instrument). The instrument was configured with RTV-specific physicochemical parameters derived from micrometer and submicrometer particle characteristics. For the former, a refractive index of 1.5 and absorption index of 0 were used,^21^ while for the latter, experimentally determined values were used, i.e., a refractive index of 1.4 for both red and blue lasers, with corresponding absorption indices of 0.001 and 0.01, respectively.

Ultrapure water (refractive index of 1.33) was used as the dispersing medium in the instrument’s dispersion unit. Background measurements were acquired before sample analysis to establish baseline readings. Following sample preparation (as detailed in Sections 2.3, 2.4 and 2.5), suspensions were added into the dispersion unit, maintaining constant agitation at 2000 rpm, until target obscuration values (light transmission loss) of approximately 6% for particles ≥1 μm and approximately 10% for particles <1 mm were achieved. Continuous agitation at 2000 rpm was maintained throughout the measurements to ensure a uniform particle dispersion. Size distribution percentiles (D10, D50, and D90) and the polydispersity value (span index) were monitored throughout the characterization process.

#### 2.6.2 Particle Size Distribution Determination by Dynamic Light Scattering

Once the particles exhibited D90 values approaching 1 μm, dynamic light scattering (DLS) analyses were also performed, as this analytical technique is optimized for submicrometer particle size determination.

Using the Zetasizer Nano ZS90 (Malvern), the average particle size (*Z*-average) and polydispersity index (PDI) were determined. Sample preparation involved a 1:1000 dilution, wherein 10 μL of processed material was volumetrically diluted to 10 mL using ultrapure water as the dispersing medium. All measurements were conducted at room temperature. Colloidal stability was assessed concurrently through zeta potential (mV) measurements using the same instrument. The analysis used the RTV-specific physicochemical parameters as previously described in Section 2.7.1.^21^

#### 2.6.3 X-ray Diffraction

The diffraction measurements were carried out using a Shimadzu X-ray diffractometer, model XRD-6100, with CoKα radiation (λ = 1.54 Å), tube current, and voltage set at 30 mA and 40 kV, respectively. The angles were explored from 3° to 40° in 2θ, with a step size of 0.2° per minute.

The crystal structures of the RTV forms (form I and form II) were resolved by Bauer and collaborators (2001) and deposited in the Cambridge Structural Database.^22^ Access was obtained through the Cambridge Crystallographic Data Centre (CCDC) Web site, deposition number 710528.

#### 2.6.4 Thermogravimetric Analysis

Using a TA Instruments thermogravimetric analyzer, model TGA 5500, the samples were subjected to a temperature range of 20 to 600 °C with a heating rate of 20 °C/min. Nitrogen was used as the purge gas at a flow rate of 50 mL/min.

#### 2.6.5 Differential Scanning Calorimetry

Approximately 5 mg of each sample was carefully weighed into sealed aluminum pans and heated at 20 °C/min to 200 °C, under a nitrogen atmosphere flowing at 50 mL/min. The analysis was conducted using a differential scanning calorimeter (TA Instruments, model DSC250).

#### 2.6.6 Hot-Stage Microscopy

Hot-stage microscopy (HSM) and polarized light optical microscopy were performed by using a Leica DM2700-M microscope equipped with a Linkam LTS420 heating stage (Leica Microsystems, Wetzlar, Germany) and a Leica MC170 HD camera. Samples were deposited on a glass slide and heated from room temperature to 200 °C at a rate of 10 °C/min. Images and videos were recorded under cross-polarized light using 50× magnification.

#### 2.6.7 Infrared Spectroscopy

Infrared spectroscopy was performed using a Thermo Scientific Nicolet iS50 FTIR spectrometer equipped with OMNIC version 7.0 software. Small quantities of the sample were placed in direct contact with the crystal of the attenuated total reflectance (ATR) accessory. The spectra were recorded from 4000 to 640 cm^–1^ with a resolution of 4 cm^–1^ and 32 scans. The spectra were acquired in percentage transmittance (%T).

#### 2.6.8 Scanning Electron Microscopy

The images were obtained using a Tescan LYRA3 scanning electron microscope with an electron beam of 5 kV. Small amounts of the sample were adhered to double-sided adhesive tape placed on a smooth support. The samples were then sputter-coated with a thin layer of gold at room temperature under an argon atmosphere and high vacuum for subsequent evaluation. The sputter coater used was model 108 from Cressington.

#### 2.6.9 Dissolution Test (In Vitro Evaluation)

For the dissolution of RTV nanocrystals, nanosuspension, and raw RTV API, 100 mg of RTV, corrected by sample potency, were added to dissolution vessels (in triplicate) containing 900 mL of POE10LE 0.06 M dissolution medium. The equipment used was a Distek Evolution 6100 dissolution tester. The system temperature was set to 37 °C, and the dissolution apparatus was paddle type (apparatus 2) with a rotation speed of 75 rpm. The selected parameters were based on the USP monograph for the oral solid dosage form of RTV.^23^ After the dissolution began, 5 mL aliquots were collected at 5, 10, 15, 30, 45, 60, 90, 120, 150, and 180 min, with no medium replenishment. The aliquots were filtered using a syringe filter with a filter membrane pore size of 0.1 μm made of PTFE, and the readings were taken with a Shimadzu spectrophotometer, model 1900i, at a wavelength of 240 nm. The final RTV concentration values were obtained from previously constructed calibration curves, evaluated for linearity, and analysis of variance (ANOVA) using Excel software (Microsoft).

Based on the dissolution profiles, calculations were performed to obtain dissolution efficiency (DE) values, where the ratio between the area under the RTV dissolution curve, within the time range from zero to 90 min (0–90 min), and the total area of the rectangle defined by the ordinate (100% dissolution) and the abscissa (time equal to 90 min) was used.^24^

#### 2.6.10 In Vivo Biodistribution: Tissue Deposition

##### 2.6.10.1 Radiolabeling Process with [^99^mTc] of Both: Nanocrystal and Nanosuspension

The radiolabeling process with technetium-99m ([^99^mTc]) was initially performed by adding 0.3 mL of stannous chloride (SnCl_2_) at a concentration of 80 mg/mL to 0.6 mL of [^99^mTc] with an activity of 6.9 mCi, followed by a 10 min incubation. Subsequently, the radiolabeling was conducted separately for each compound, using 50 mg of nanocrystals and an equal amount of nanosuspension, both of which were incubated with [^99^mTc] to achieve the radiolabeling of the structures.

##### 2.6.10.2 Quality Control of Radiolabeling with [99 mTc]

To confirm the efficiency of the labeling process, the thin-layer chromatography (RTLC) technique was employed. For this purpose, 2 μL of the radiolabeled nanosystems were applied to Whatman No. 1 paper, with acetone as the mobile phase. Triplicate strips were prepared to assess labeling stability over time (0, 1, 3, 5, and 24 h postlabeling). Radioactivity was measured using a gamma counter (Hidex, Turku, Finland).

##### 2.6.10.3 Animal

The experiments were conducted on male Swiss mice (*n* = 3) with an average weight of 25–30 g. The animals were housed in a controlled environment under appropriate lighting conditions (12:12 h light–dark cycle) and temperature (21.0 ± 1.0 °C). Additionally, they had free access to food and water. All procedures were approved by the Animal Experimentation Committee of the Rio de Janeiro State University (Rio de Janeiro, RJ, Brazil; Protocol CEUA/8059100220/2021) in accordance with the Guide for the Care and Use of Laboratory Animals (National Research Council, 1996), published by the United States National Institutes of Health. Finally, the animals were euthanized by an overdose of anesthesia, administered via intraperitoneal injection of ketamine (100 mg·kg^–1^) and xylazine (20 mg·kg^–1^).^25^

##### 2.6.10.4 Experimental Design

For biodistribution/tissue deposition analysis, 1.53 mCi (56.6 MBq)/0.2 mL of radiolabeled nanocrystals or nanosuspension (11.1 mg) with ^99m^Tc were administered via intraperitoneal (I.P.) injection. The animals were sacrificed 24 h after injection using an overdose of anesthesia (isoflurane). Blood and organs were immediately collected, including the brain, heart, lungs (left and right), liver, stomach, kidneys (left and right), spleen, intestines (large and small), and bladder, for the subsequent measurement using a Gamma counter (Hidex, Turku, Finland). The results were expressed as the percentage of the injected dose per gram of tissue (%ID/g).

##### 2.6.10.5 Pharmacokinetic

For pharmacokinetic analysis, 2 μL of blood was collected from the caudal vein at predefined time points (0, 2, 4, 6, 21, 23, and 24 h) following the injection of the [^99^mTc]-labeled systems. The blood samples were measured using a Gamma counter (Hidex, Turku, Finland). It is important to notice that a reliable terminal phase could not be established beyond 24 h; therefore, was not reported AUC0-∞ to avoid high extrapolated fractions.

#### 2.6.11 Biochemical Analysis

For biochemical analysis, healthy animals were administered 11.1 mg of both ritonavir-based systems via intraperitoneal (I.P.) injection. Blood samples were collected posteuthanasia, without direct radiation exposure, through cardiac puncture 24 h after nanosystem administration (nanocrystals and nanosuspension). A total of 0.5 mL of blood was drawn and mixed with 0.3 mL of an anticoagulant (heparin, Sigma-Aldrich), followed by centrifugation at 5000 rpm for 5 min at 4 °C. The plasma was then isolated, and the activity of the following biomarkers was assessed: alanine aminotransferase (ALT), aspartate aminotransferase (AST), gamma-glutamyl transferase (GGT), creatinine (CRE), cholesterol (CHOL), glucose (GLU), amylase (AMS), and lipase (LPS).

#### 2.6.12 Statistical Analysis

The in vivo experiments were performed in triplicate and expressed as mean ± SEM. Statistical differences were analyzed using GraphPad Prism 8.1 software, and a One-way ANOVA followed by the Tukey test (multiple comparisons) was performed. A *p*-value of ≤0.05 was adopted and was considered statistically significant.

## 3 Results and Discussion

### 3.1 Sample Processing with Ultra-Turrax Tube Drive Disperser

#### 3.1.1 Evaluation of the Milling Progress with Different Bead Sizes

The data from the bead size influence on nanosuspension milling (Section 2.3.1) using an Ultra-Turrax Tube Drive disperser are presented in Table 1 and Supporting Information.

**Table 1:** Comparison between all Milling Combinations Used

	LD				DLS		
sample	D10 (μm)	D50 (μm)	D90 (μm)	span	*Z*-Ave (nm)	PDI	ZP (mV)
only one type of beads							
500 μm	0.201	0.410	0.843	1.568	396.7	0.166	–36.8
200 μm	0.152	0.310	0.585	1.397	300.7	0.170	–30.0
100 μm	0.267	0.559	1.390	2.013	533.6	0.253	–40.8
beads combination							
500 μm + 200 μm	0.168	0.334	0.626	1.372	302.5	0.156	–36.3
500 μm + 100 μm	0.170	0.342	0.641	1.378	304.1	0.134	–35.2
combination of TURRAX/beads							
TURRAX +500 μm	0.212	0.427	0.862	1.525	378.3	0.218	–37.7

By comparing the D50 and *Z*-ave values, especially in the Supporting Information, it is possible to infer that both techniques, DLS and LD demonstrated good correlation and complementarity, supporting the finding that all bead sizes tested successfully performed the milling of RTV, leading it to the nanometric scale. Nevertheless, the effectiveness of milling process differed with the bead size.

As for the zeta potential, similar values were obtained in all cases, indicating that the bead size may influence RTV milling but did affect significantly the particle charge. Moreover, the values > 30 mV suggests good stability according to the classical DLVO theory.^26^

Concerning the use of the Ultra-Turrax T25 disperser, a test was performed to assess the equipment’s ability to reduce the particle size of the RTV API. The test lasted 15 min, and the equipment was set at a constant speed of 11,000 rpm. The disperser in question was not able to significantly reduce the D10, D50, and D90 values. Even the polydispersity value remained unchanged.

However, this did not completely rule out the use of Ultra-Turrax in a process as it could facilitate future scale-ups. To perform such a test, and based on the values obtained in Table 1, the Ultra-Turrax was used prior to the milling process with 500 μm beads. The results are presented in Table 1, where it is observed that during the use of the TURRAX, even at a higher rotation speed (18,000 rpm), no milling improvement was achieved.

To facilitate the evaluation of the different experiments, a graph was created to assess the performance of the grinding media over time, compiling the D50 and *Z*-ave values presented in Table 1 (final time) together with the monitoring data collected every 30 min, which are presented in the Supporting Information. See Figure 2.

**Figure 2:**
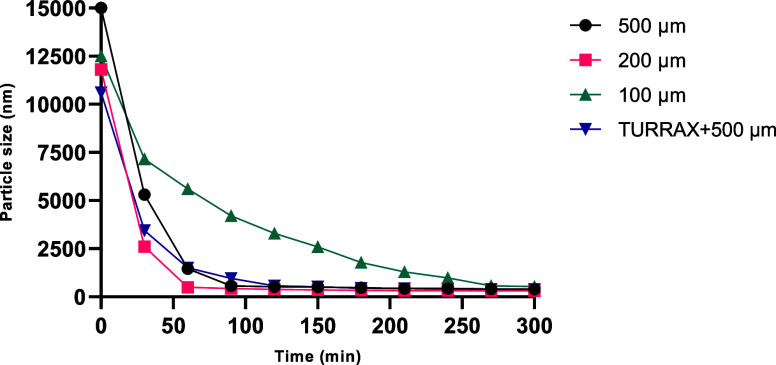
Milling progress using different bead sizes and TURRAX in the milling of presuspensions.

According to the results presented in Figure 2, it is evident that the processes using 200 and 500 μm beads, with or without TURRAX, demonstrated the best performances compared to milling with 100 μm beads. In the case of the isolated use of 100 μm beads, performance was lower, especially when the values obtained at the end of each process are considered (Table 1), where the size values (D50) were also the highest. This effect was attributed to the lower weight displayed by the 100 μm beads when compared to the others. This characteristic may result in lower energy in the milling process, leading to less efficiency in the comminution process.

Finally, combinations of bead sizes were tested, where the initial milling was performed with larger beads (500 μm) for 4 h, and after this time, the nanosuspension was separated by decantation from the original beads and transferred to another Tube Drive with smaller beads (200 or 100 μm) (Table 1).

Initially, it is observed that the D50 and Z-ave results throughout the processes showed good correspondence and similarity. Regarding the D10, D50, D90, and *Z*-ave sizes obtained at the end of the processes, all tested combinations successfully milled the RTV, reaching the nanometric scale.

In this specific case, the 100 μm beads achieved better performance compared to their previous result (Table 1), primarily because they were already processing particles with a significantly reduced size (approximately 400 nm in diameter), as can be observed in the Supporting Information. As for the zeta potential, similar values were obtained in all cases.

When analyzing all aspects of Table 1, it is evident that the use of 200 μm beads showed the best performance, but the use of 500 μm beads should not be dismissed. Despite presenting slightly higher D50 and *z*-average values as well as larger polydispersions, these beads may be promising, particularly when considering the use of more powerful equipment with greater capacity for producing RTV nanocrystals. This is especially relevant given the lower cost of acquiring larger beads and their easier handling, especially regarding the recovery of the nanosuspension (NSP). The combination of beads also produced good results but nothing that justifies its use over the results described earlier, especially considering the greater difficulty of operationalizing bead exchanges on a larger scale. Only the isolated use of 100 μm beads proved to be an unviable option.

#### 3.1.2 Sample Processing with Selection of Steric Stabilizers

According to the results presented in Section 3.1.1, the proportions of SLS and RTV to be used in the presuspensions were confirmed, and 500 μm beads were chosen for use. The choice of 500 μm beads was due to their ease of handling and the fact that their results yielded a particle size scale relatively close to those achieved with 200 μm beads (Table 1). Thus, the results involving the selection of different steric stabilizers are compiled in Table 2, where initially promising results were obtained with the following materials: HPC LF, HPC SSL, HPMC, P188, and P407. However, it was observed that NSPs produced with HPC LF, P188, and P407 had higher D90 values when the LD technique was used, which resulted in a proportional increase in dispersion (span). Although the LD technique cannot measure larger particles, the equipment detects the presence of such elements, and a similar effect is reflected in their dispersion values (PDI), which were higher when these stabilizers.

**Table 2:** Comparison of Results in the Milling (300 min) of Presuspensions with Different Steric Stabilizers

	LD				DLS		
sample	D10 (μm)	D50 (μm)	D90 (μm)	span	*Z*-Ave (nm)	PDI	ZP (mV)
PVP	0.201	0.410	0.843	1.568	396.7	0.166	–36.8
HPC LF	0.205	0.428	0.929	1.689	472.5	0.229	–33.4
HPC SSL	0.196	0.399	0.794	1.500	351.3	0.205	–38.0
HPMC	0.090	0.272	0.733	2.365	377.0	0.239	–34.6
P188	0.235	0.483	1.590	2.815	494.3	0.265	–33.2
P407	0.234	0.482	1.470	2.555	519.5	0.273	–32.1
SSG	5.170	135.000	620.000	4.546	-	-	-
CCS	2.720	25.200	1930.000	76.424	-	-	-

The results of the milling progress over time are provided in the Supporting Information.

Different results were obtained with the use of sodium starch glycolate (SSG) and croscarmellose sodium (CCS), substances considered suspension adjuvants with stabilizing effects, not only steric but also with kinetic characteristics.^27^ In both cases, more viscous presuspensions were formed, and greater difficulty was observed in dissolving both stabilizers in water, requiring system heating.

Milling the samples with SSG and CCS was halted after 60 min of processing, as possible particle agglomerations occurred in the system, resulting in samples with larger dimensions than those presented at the beginning of the process (unmilled raw material).

Finally, after analyzing the data related to sample processing, including bead size variation (Table 1), bead size combinations (Table 1), and variation of steric stabilizers (Table 2), along with what has already been discussed, considerations regarding processing times and polydispersity results are appropriate. It is noted that there is a sharp decrease in the average particle size, especially in the first hour (Figure 2), and after this point, further improvement mainly involves a finer reduction in size and better polydispersity results. In nearly all cases, the process can be stopped after 4 or 5 h and continuing would be more related to achieving a specific target value, with particle sizes smaller than 300 nm becoming highly unlikely, possibly indicating an intrinsic limit to the RTV crystals.

### 3.2 Scaling-Up Formulations with DeltaVita 300 Bead Mill

This study, based on the results from Sections 3.1.1 and 3.1.2, entered the scale-up stage using the DeltaVita 300 bead mill. The previous stage focused on screening the best conditions for obtaining nanosuspensions. However, due to the small volumes processed, spray drying would not have been feasible as it requires a larger sample volume. Additionally, the strategy established considered this stage as providing information for scaling-up with fewer parameter variations, thus saving on the use of the API, which is of high value. Although the stability of all systems was guaranteed for up to 7 days during small volume batches, spray drying was performed immediately after production of each nanosuspension batch.

As mentioned in Section 2.4, the batches were produced with a total volume of 500 mL, using 500 μm beads, and maintained a 10% drug load (w/v), 0.2% charge stabilizer (w/v), and 0.5% steric stabilizer.^20^ Although the Ultra-Turrax T25 disperser did not show any influence on particle size reduction, it was used to produce more homogeneous suspensions, which is beneficial during batch scale-up.

Initially, two batches were produced to test the influence of the mill’s rotor speed. One batch, after passing through the Ultra-Turrax T25 disperser, underwent 10 min of milling at 1000 rpm and then continued for 120 min at 2000 rpm.^20^ The second batch differed in that the milling at 1000 rpm lasted for the entire 120 min period. Both batches used PVP K30 as the steric stabilizer, as it had shown good results in previous tests.

Subsequently, a third batch was produced by milling at 1000 rpm for the full 120 min, but this time HPMC was used as the steric stabilizer. HPMC was another steric stabilizer that yielded good milling results, as shown in Section 3.1.2. The results of the bead milling process are compiled in Table 3.

**Table 3:** Comparison of the Milling Results of Nanosuspensions Produced with the DeltaVita 300 Bead Mill

	LD				DLS		
sample	D10 (μm)	D50 (μm)	D90 (μm)	span	*Z*-Ave (nm)	PDI	ZP (mV)
milling at 2000 rpm with PVP K30							
unmilled sample	4.650	11.600	46.500	4.650	-	-	-
30 min milling	0.190	0.395	0.863	1.706	328.3	0.211	–32.3
60 min milling	0.163	0.341	0.672	1.492	301.3	0.171	–29.9
90 min milling	0.145	0.305	0.612	1.531	293.9	0.195	–30.8
120 min milling	0.142	0.300	0.613	1.566	294.5	0.188	–32.2
150 min milling	0.131	0.277	0.574	1.602	289.1	0.182	–32.8
milling at 1000 rpm with PVP K30							
unmilled sample	4.480	12.300	943.000	76.232	-	-	-
30 min milling	0.029	0.144	1.090	7.338	334.4	0.196	–26.1
60 min milling	0.169	0.349	0.688	1.486	293.5	0.206	–40.9
90 min milling	0.150	0.313	0.616	1.493	269.3	0.191	–36.6
120 min milling	0.144	0.301	0.579	1.443	259.4	0.181	–30.5
milling at 1000 rpm with HPMC							
unmilled sample	4.570	13.200	814.000	1.749	-	-	-
30 min milling	0.207	0.425	0.950	1.551	354.0	0.225	–37.4
60 min milling	0.183	0.374	0.763	1.751	290.6	0.215	–34.1
90 min milling	0.188	0.392	0.874	1.784	268.8	0.172	–34.3
120 min milling	0.155	0.320	0.641	1.519	259.7	0.173	–29.6

Given the results presented earlier, it can be concluded that the scaling up of batches through milling with the DeltaVita 300 bead mill was successfully achieved and reflected the preliminary progress obtained in the batches produced using an Ultra-Turrax Tube Drive disperser.

The final particle size values (D10, D50, D90, and *z*-average) were similar to the final values resulting from the Tube Drive but longer milling times (4 to 5 h) were required in the Tube Drive for the D50/*z*-average value to reach approximately 300 nm. In contrast, these values were obtained in just 30 min with the bead mill. This is a significant finding and highlights the high energy provided by the mill, particularly because all of the components of the milling chamber (beads, rotor, and chamber walls) are made of zirconium oxide, which provides multiple points of impact and milling. In the Tube Drive, only the beads are made of zirconium, with the loading container made of plastic, allowing more effective milling only during the bead collisions.

Despite the structural differences between the pieces of equipment, their relationship allowed for various earlier tests (small batches) to be conducted without the high material cost required to operate the minimum batch size in the DeltaVita mill. To give a clearer comparison, each batch produced in the Tube Drive used 1 g of RTV, while in the bead mill, the minimum amount of RTV was 50 g, a mass 50 times larger. This is highly relevant, especially considering the high cost of the RTV API.

Regarding rotor speed, similar values were obtained with both 2000 and 1000 rpm, producing similar particle sizes in just 60 min of processing.

### 3.3 Drying of Nanosuspensions via a Spray-Drying Technique

The materials produced during the formulation scale-up using the DeltaVita 300 bead mill (Section 3.2) were subjected to drying by the spray-drying technique and regardless of the type of sample (Table 3)-whether the sample processed at a higher rotation speed (2000 rpm), the one processed at a lower rotation speed (1000 rpm), both using PVP K30 as the steric stabilizer, or the sample using HPMC at 1000 rpm, all displayed the same behavior during the drying process.

The nanosuspensions, after recovering from the mill, were placed in a beaker under constant agitation and passed through the spray-dryer system, with parameters set according to Section 2.5. Several important observations were made after the process was completed. Initially, material accumulation was observed in the cylinder of the equipment; this material formed a smooth homogeneous layer on the glass, which was collected for further characterization.

Supporting the previous description, it was also noted that the yield of the dried material did not reach a high percentage, achieving only around 10% of the RTV mass added to the presuspension. One alternative to improving the yield was to vary the atomizer nozzle temperature between 120 and 140 °C, but no significant changes in yield or process characteristics were observed in any of these cases.

The final material obtained was a white finely divided powder with significant flowability. It is important to note that all of the dried samples produced during this stage were subjected to characterization by IR, DSC, TG, XRD, and SEM to observe any potential morphological or crystalline changes. The material collected from the cylinder of the equipment was also subjected to these characterizations, and the results are presented in Section 3.4.

### 3.4 Sample Characterization

#### 3.4.1 X-Ray Diffraction

The diffractograms of the RTV API and the samples dried in the spray-dryer can be seen in Figure 3. According to the presented image, it is possible to confirm the crystalline nature of all of them, which are representations of polymorph II (orthorhombic crystal system), as per the literature data. Their diffractograms show many reflections; however, according to the literature,^28^ form I can be characterized by the presence of two peaks at 2θ values of 3.32° and 6.75°, which were not found in the diffractogram of the RTV API or in any of the spray-dried samples. Form II, on the other hand, has characteristic peaks at 9.51°, 9.88°, and 22.2°, all of which are present in the diffractograms shown.^22^

**Figure 3:**
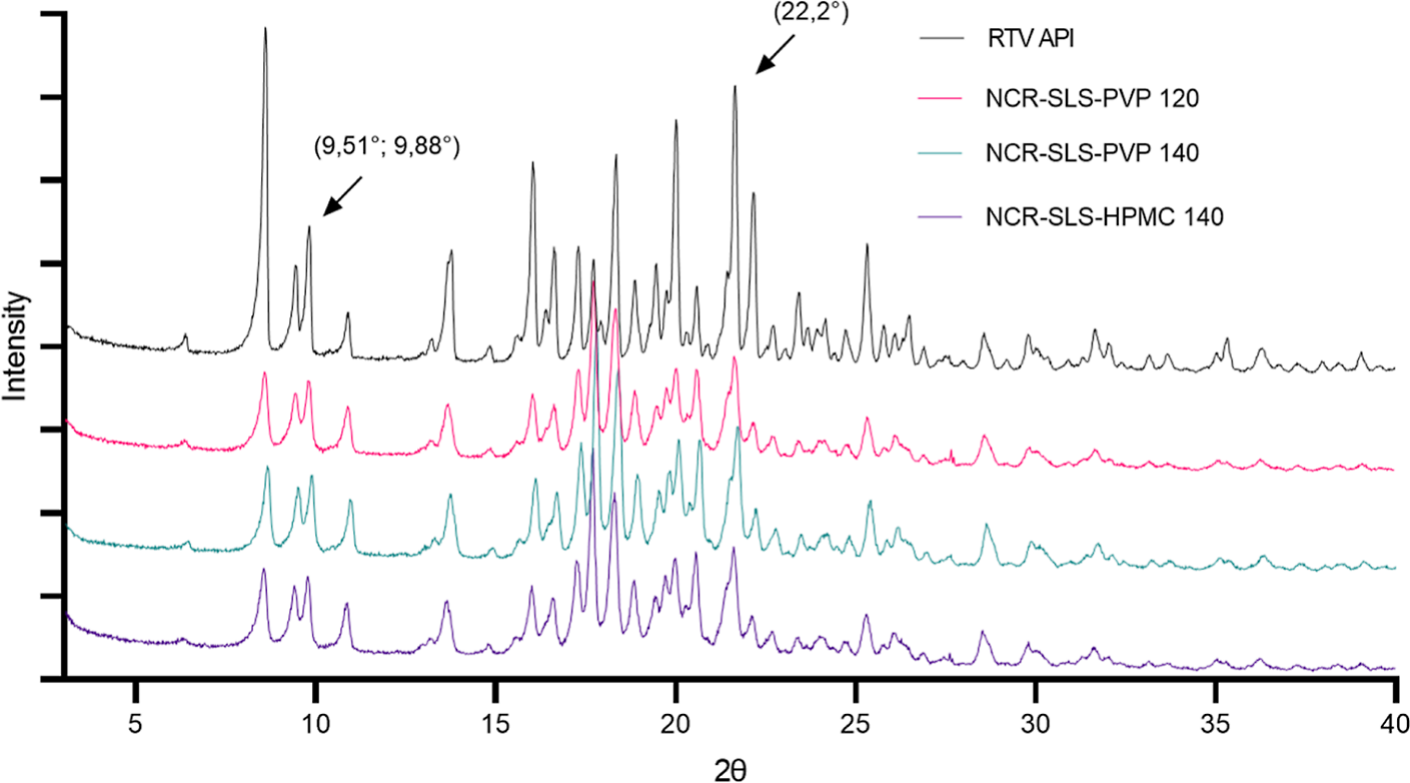
RTV API and processed samples diffractograms.

It is very important to emphasize that the RTV crystals subjected to the drying process maintained their crystalline characteristics, without showing any phase transition to form I. However, it is inevitable that the obtained nanocrystals may exhibit some reduction in crystallinity, as the peak intensities in the processed samples showed some smoothing.^29^

#### 3.4.2 Thermogravimetric Analysis

The TGA analysis was performed to evaluate the thermal behavior of RTV API in comparison to the other formulations after spray-drying. Mass losses (moisture content and decomposition temperatures) and the thermal stability of RTV were analyzed.

The mass loss of RTV API and the samples can be seen in Figure 4, where a first event is observed at approximately 200 °C, and, later, a second event near 300 °C. These two events likely correspond to the decomposition process of the sample, as reported in the literature.^22,28^

**Figure 4:**
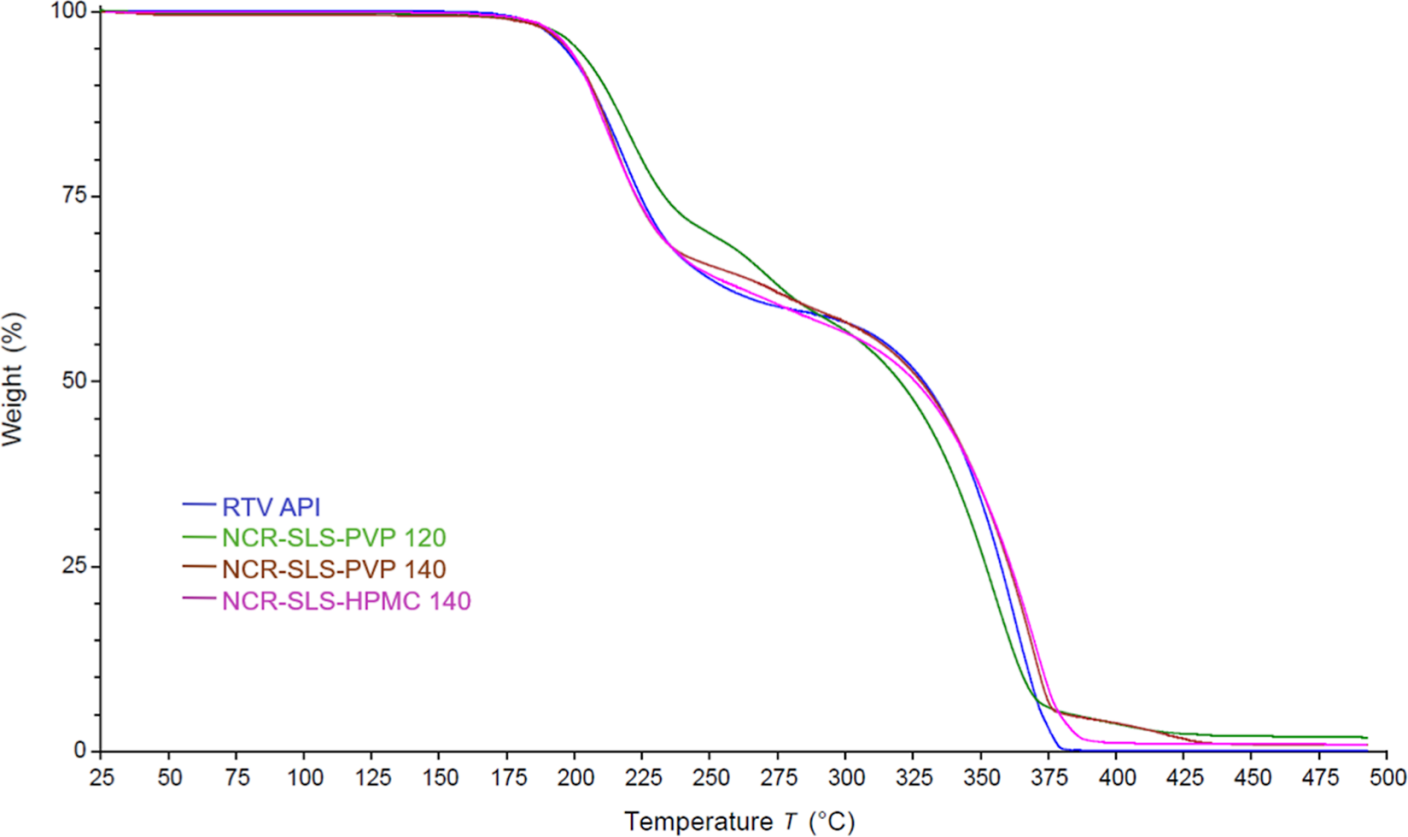
TGA curves of RTV API and processed samples.

It is important to note that these stages do not provide insights into the crystalline form, as the TGA profile shows few events and is very similar across the various polymorphic forms.^28,30^

#### 3.4.3 Differential Scanning Calorimetry

In addition to the TGA analyses, the DSC curves of RTV API and the samples dried by spray-drying can be seen in Figure 5. As presented, the DSC curve of RTV API showed a sharp endothermic peak around 129 °C. According to the literature, this event is associated with the drug’s melting point.^28,30^ No other events were observed. The same endothermic event can be seen in the curves of the spray-dried samples and the sample retained in the cylinder. In these cases, a slight shift in the melting point was observed, possibly caused by the introduction of stabilizers into the systems (SLS, PVP K30, and HPMC), which is something expected when dealing with mixtures, or alternatively, due to the potential loss of crystallinity observed in the diffractogram of the samples (Figure 5).^31,32^

**Figure 5:**
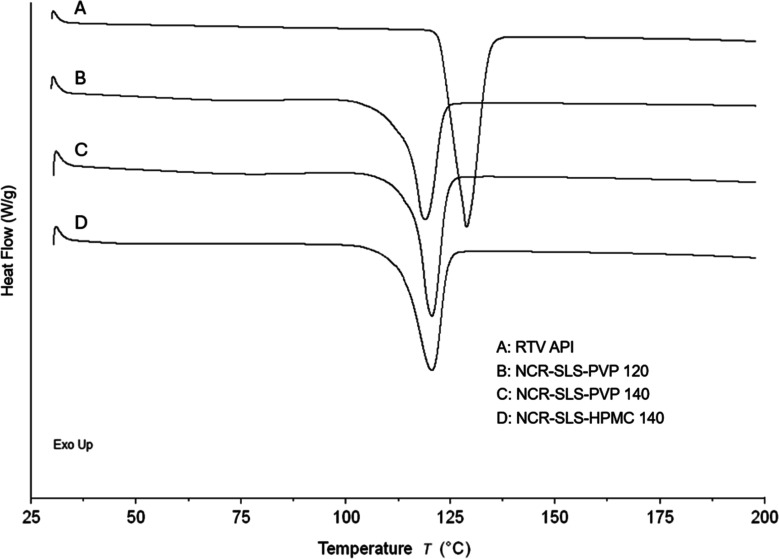
DSC curves of RTV API and processed samples.

To better understand these thermal changes, the degree of crystallinity of the samples was estimated based on their fusion enthalpies. The RTV API exhibited a fusion enthalpy of 123.78 J/g and was therefore used as the reference for a fully crystalline Form II material. After the correction of the drug content in the formulations (93%), the nanocrystal samples presented fusion enthalpies of 68.46 J/g (NCR-SLS-PVP 120), 80.29 J/g (NCR-SLS-PVP 140), and 71.91 J/g (NCR-SLS-HPMC 140). These values correspond to relative crystallinities of 77.3%, 90.6%, and 81.1%, respectively, demonstrating that the milling process induced a measurable reduction in the crystalline fraction. This quantitative reduction is consistent with the broadening and slight depression of the melting endotherm observed in the DSC profiles and agrees with the PXRD data, where the characteristic reflections of Form II are preserved but appear to be less intense. Together, these results confirm that the nanocrystals retain their polymorphic identity while exhibiting partial amorphization and lattice disorder, phenomena commonly reported for nanosized drug systems.^20,33^

As with the TGA of RTV, these results do not provide insights into the crystalline form type, as the DSC profile shows few events and is very similar across different polymorphs.^28,30^ Therefore, these evaluations are more commonly used for monitoring the solid phase during the process than for structure resolution.

#### 3.4.4 Hot-Stage Microscopy

HSM was used to confirm the melting point observed in the DSC analysis. The thermomicroscopic analysis showed the visual melting of the crystalline material beginning at approximately 126 °C and completing around 134 °C, in agreement with the melting event detected by DSC (Figure 6). This corroborates that the endothermic peak corresponds to the melting process of ritonavir Form II.

**Figure 6:**
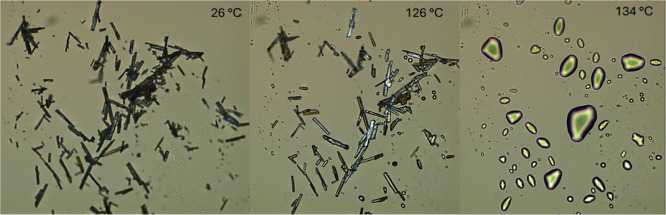
Hot-stage microscopy (HSM) images of ritonavir API under cross-polarized light showing the melting process. 26 °C initial state of the crystalline material; 126 °C onset of melting; 134 °C-complete melting of the crystals.

#### 3.4.5 Infrared Spectroscopy (FTIR)

The FTIR spectrum of RTV API, as well as the processed samples, with the main bands indicated, is shown in Figure 7. The characteristic peaks correspond to the stretching of the amide group at 3321 cm^–1^, the acidic hydrogen bond within the molecule at 2958 cm^–1^, the ester bond at 1701 cm^–1^, and the stretching of aromatic carbons at 1659 cm^–1^, 1608 cm^–1^, and 1534 cm^–1^.^34,35^ These results ruled out possible interactions between RTV and its stabilizers, as the spectrograms presented are identical in all cases.

**Figure 7:**
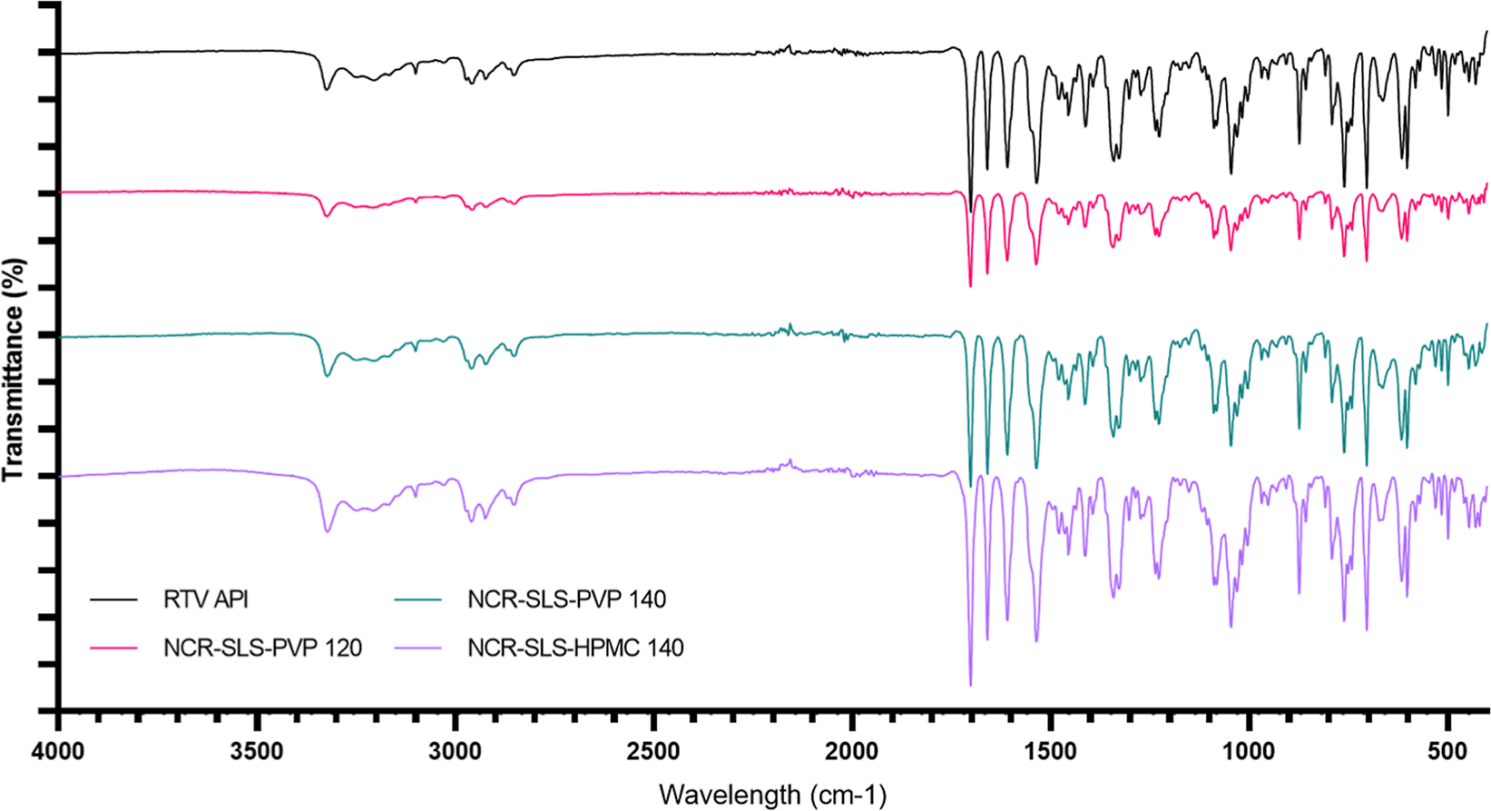
FTIR of RTV API and processed samples.

#### 3.4.6 Scanning Electron Microscopy

After the drying procedure, in all of the samples produced in Section 3.3, the presence of microparticles predominantly in the shapes of spheres and toroidal was observed. These shapes are commonly obtained in materials produced by the spray-dryer technique, where microdroplets, produced by the atomizer, are dried at ambient temperature, forming spherical microparticles (B). However, the drying of the microdroplets can occur turbulently due to carrier gas, causing variations in the shape and size of the dried particles.^36,37^ Regarding the toroidal geometry (C), as the drying of the droplets progresses, the nanocrystals accumulate at the air interface, and a solid shell forms due to Darcy’s pressure. If this pressure becomes critical, the solvent flow through the shell can rupture it, leading to these formations.^36,38^

Figure 8 presents the SEM image of RTV API at a magnification of ×1500 (A), as well as a processed and spray-dried sample at different magnifications (×1500 B, ×10000 C, and ×30000 D).

**Figure 8:**
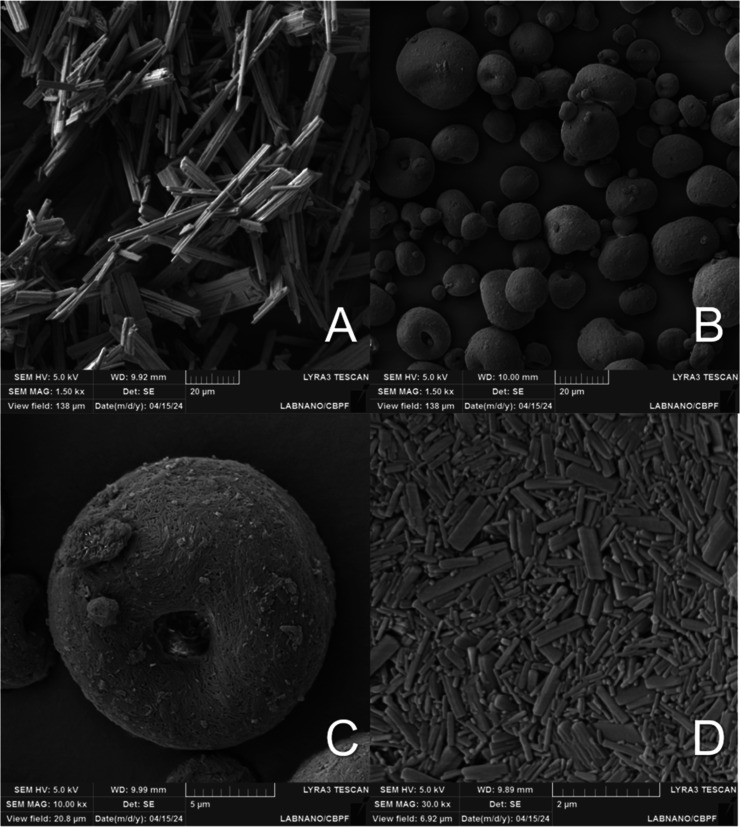
SEM of RTV API and processed sample. (A): RTV API magnification ×1500; (B): RTV nanoaggregate magnification ×1500; (C): RTV nanoaggregate magnification ×10000; and (D): RTV nanoaggregate surface magnification ×30000.

Regarding the RTV API, this morphology, although only an indicator of the API’s crystalline form, is consistent with what is expected for form II, according to the literature,^22,28^ demonstrating the presence of rod-shaped or bar-shaped crystals (A).

In relation to the processed samples, the obtained microparticles are of the nanoaggregate type,^38^ as, after selecting a single unit (C) and increasing the magnification, the presence of crystals with at least one dimension on a nanoscale is observed after drying (D).^39,40^

#### 3.4.7 Dissolution Test (In Vitro Evaluation)

To comparatively evaluate the in vitro performance of the nanoparticles produced in the previous stages (Sections 3.2 and 3.3), two spray-dried samples at 140 °C (NCR-SLS-PVP and NCR-SLS-HPMC), the nanosuspension before drying (NSP-SLS-PVP), and the unprocessed RTV API were selected for the dissolution test. The results obtained using POE10LE at 0.06 M as the dissolution medium are presented in Figure 9.

**Figure 9:**
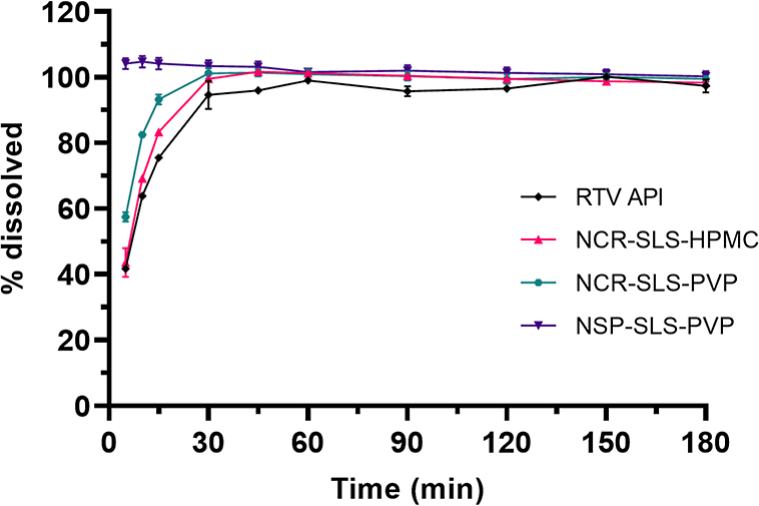
Dissolution of RTV API and processed samples in POE10LE 0.06M.

When the dissolution profiles of the processed samples were compared with the RTV API, it can initially be observed that the nanocrystals of RTV in the suspension have a high dissolution capacity, as maximum dissolution was achieved in the initial minutes. Regarding the spray-dried samples, an improvement in dissolution compared to the API is noted, especially in the first 30 min; however, the improvement is not very pronounced. This performance may have been hindered by the characteristics of the dried materials, which show finely divided powders with low density, making wettability and dispersion in the dissolution medium more difficult. In contrast, API was dispersed immediately in the dissolution medium.

To gain further insights into this matter, a new dissolution test was conducted using a more discriminative medium, i.e., one with a lower dissolution capacity. For this stage, a POE10LE of 0.04 M was chosen, whose the surfactant concentration, combined with a volume of 900 mL and a mass of 100 mg of RTV, created a system close to its saturation point. The results are presented in Figure 10.

**Figure 10:**
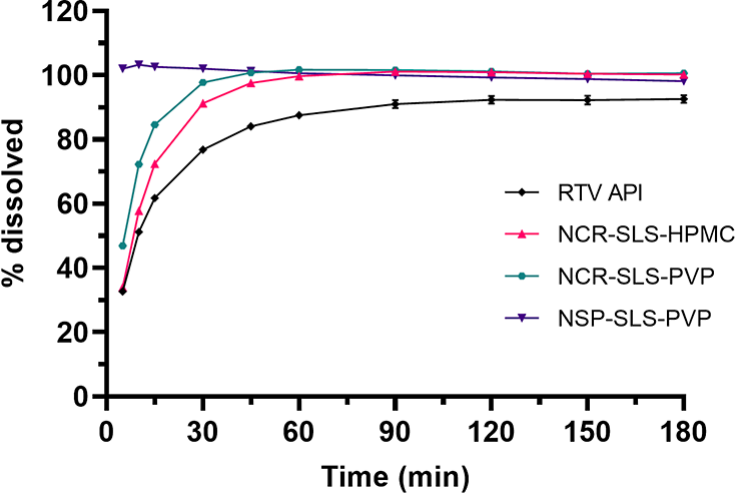
Dissolution of RTV API and processed samples in POE10LE 0.04M.

In this second paper, a clear distinction can be observed between the RTV API and the spray-dried samples. While the NCR-SLS-PVP and NCR-SLS-HPMC nanocrystals still maintained a good dissolution profile, the RTV API showed a decline, failing to reach 100% dissolution even after 180 min of testing. This effect is due to the proximity to the system’s saturation limit, which makes it more difficult for micrometric samples to dissolve compared to nanometric particles. Much of this effect is attributed to supersaturation effects and increased surface area achieved through reduction to the nanometer scale, which is evident when observing the nanosuspension profile.

Regarding the spray-dried samples, they needed to disintegrate from their nanoaggregate state before achieving complete dissolution. Even so, it is noteworthy that the performance of the spray-dried samples is significantly superior to that of the RTV API.

All of these results become even clearer when looking at the DE (dissolution efficiency) values for each medium presented in Table 4. The DE calculation was performed as described in Section 2.6.8, and the definition of the 90 min time frame was chosen because it was the time at which the RTV API reached a plateau in its worst-case scenario (POE10LE 0.04M).

**Table 4:** Comparison of Dissolution Efficiency between Samples

samples	DE (%)	SD	RSD (%)
POE10LE 0.06M			
RTV API	86.75	0.63	0.73
NCR-SLS-HPMC	91.13	0.42	0.46
NCR-SLS-PVP	93.88	1.22	1.30
NSP-SLS-PVP	100.02	1.26	1.26
POE10LE 0.04M			
RTV API	75.42	0.70	0.93
NCR-SLS-HPMC	86.40	0.36	0.42
NCR-SLS-PVP	91.46	0.30	0.33
NSP-SLS-PVP	98.50	0.53	0.54

### 3.5 In Vivo Biodistribution: Tissue Deposition

#### 3.5.1 Quality Control

The radiolabeling process was evaluated using radio-thin layer chromatography (RTLC). The results demonstrated a labeling efficiency exceeding 90% (as shown in Table 5), confirming the reliability and stability of the labeling achieved with the described method.

**Table 5:** Radiolabeling Efficiency with [^99m^Tc] as a Function of Time

time (h)	nanocrystal, %	nanosuspension, %
0	98.23	95.94
1	97.13	99.06
3	93.54	97.64
5	93.87	98.74
24	97.95	99.46

#### 3.5.2 Biodistribution and Pharmacokinetics

The biodistribution assays presented in Figure 11 and Table 6 demonstrate the uptake of both systems in the bladder, kidneys, liver, intestines, and stomach. The retention of the compounds in the kidneys up to the final time point of 24 h suggests the prolonged presence of the nanosystems, which is attributed to their negative surface charge, facilitating selectivity by the glomerular filtration barrier.^41^

**Figure 11:**
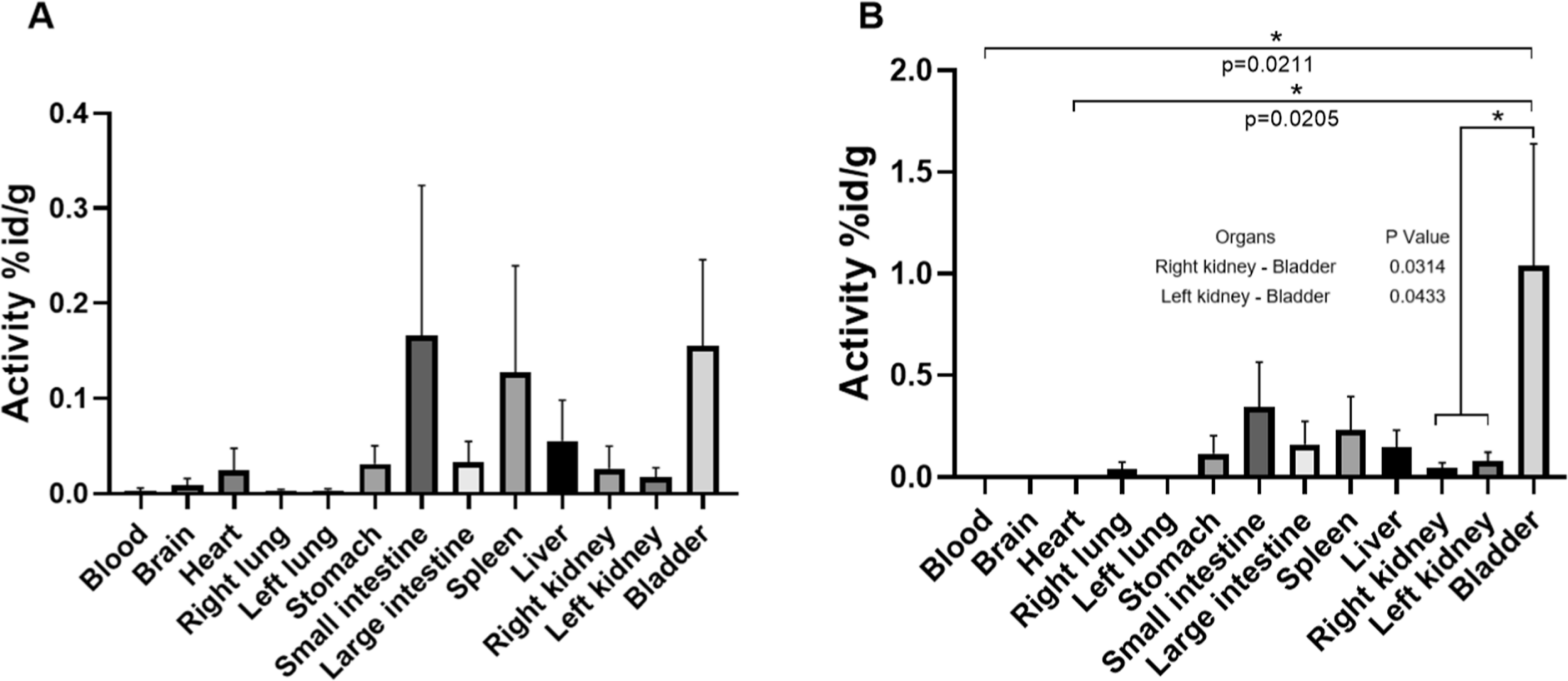
Biodistribution of ritonavir nanosystems radiolabeled with [99 mTc]. Panel A shows animals treated with nanocrystals, and panel B shows animals treated with ritonavir nanosuspension. The animals were monitored for 24 h and, at the end, euthanized for blood and organ collection. The results were performed in triplicate, and the standard error of the mean (SEM) was used. One-way ANOVA (multiple comparisons) followed by the Tukey’s test was used, and *p* < 0.05 was considered significant.

**Table 6:** Biodistribution Values of Ritonavir Nanocrystals and Nanosuspension^a^ ^,^ ^b^

nanocrystal				nanosuspension			
comparisons	*P* value	average ± SEM	significance*P < 0.05	comparisons	*P* value	average ± SEM	significance*P < 0.05
heart	-	0.02408 ± 0.02327	ns	heart	-	0.000869 ± 0.000637	ns
blood	0.9997	0.002961 ± 0.002747		blood	>0.9999	0.003820 ± 0.002158	
brain	0.9997	0.00839 ± 0.007378		brain	>0.9999	3.087 × 10^–5^ ± 3.087 × 10^–5^	
right lung	0.9997	0.003317 ± 0.001024		right lung	0.9998	0.03811 ± 0.03542	
left lung	0.9997	0.003294 ± 0.001424		left lung	>0.9999	0.004792 ± 0.003372	
stomach	0.9999	0.03129 ± 0.01883		stomach	0.9994	0.1164 ± 0.08822	
small intestine	0.5666	0.1658 ± 0.158		small intestine	0.8015	0.3458 ± 0.2178	
large intestine	0.9999	0.03273 ± 0.02207		large intestine	0.9991	0.1606 ± 0.1173	
spleen	0.8711	0.1267 ± 0.1129		spleen	0.9788	0.2332 ± 0.1626	
liver	0.9995	0.05506 ± 0.04306		liver	0.9992	0.1467 ± 0.08492	
right kidney	>0.9999	0.02627 ± 0.0235		right kidney	0.9998	0.04444 ± 0.02629	
left kidney	>0.9999	0.01726 ± 0.01004		left kidney	0.9996	0.07858 ± 0.04464	
bladder	0.6604	0.1546 ± 0.09133		bladder	0.004	1.038 ± 0.6007	**

a ns. Not significant. *p < 0.05.

b The table shows the figures obtained from the one-way ANOVA statistical analysis with multiple comparisons, where the organ of comparison was the heart. A *P*-value <0.05 was considered significant and Dunnett’s test was applied.

In the liver, nanoparticle uptake occurs via the mononuclear phagocyte system. Approximately 95% of the nanoparticles are sequestered by Kupffer cells, preventing them from reaching their intended target. However, the ritonavir nanocrystal exhibited a peak in blood concentration, as described in Figure 12, suggesting uptake by hepatocytes, which may facilitate the redistribution of the nanosystems into the bloodstream and bile.^42^ Another hypothesis to explain the previously mentioned peak is that the nanocrystal sample consists of microparticles formed by nanoaggregates (Figure 8), which undergo an initial disintegration stage to ultimately release the nanocrystals. The presence of ritonavir nanosystems in the intestines may be associated with their proximity to the injection site within the abdominal cavity.^43^

**Figure 12:**
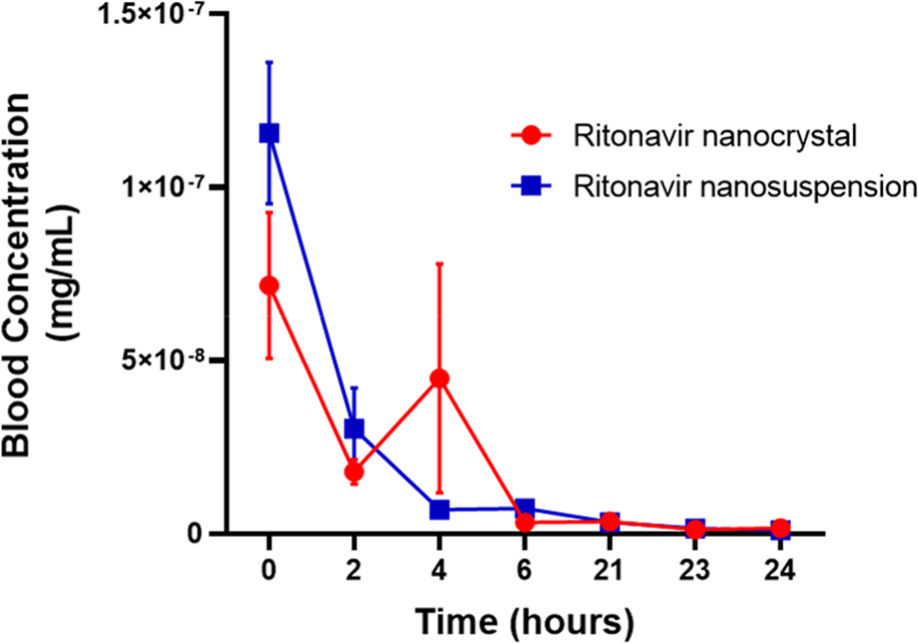
Pharmacokinetic graph of nanocrystals (red) and nanosuspension (blue) based on peripheral blood samples from male Swiss mice (*n* = 3). The standard error of the mean (SEM) was used, and measurements were taken at time points from 0 to 24 h.

However, the two systems differ in their uptake in the heart, as observed by the nanocrystals (Figure 11A). Three nonexclusive drivers for the greater myocardial signal observed with nanocrystals were hypothesized: (i) exposure-driven delivery during an early systemic peak. Spray-dried nanoaggregates can disintegrate in vivo, transiently increasing the circulating particle burden before complete dissolution; organ exposure generally scales with blood concentration–time (AUC), so a short-lived rise in systemic levels would proportionally increase delivery to highly perfused tissues such as myocardium. This exposure–uptake coupling is a standard pharmacokinetic principle;^44^ (ii) Protein-corona-mediated nanobio interactions that differ by surface chemistry. API-rich crystal surfaces with thinner steric layers are expected to form coronas distinct from excipient-coated particles; corona composition governs cellular recognition, endothelial interactions, and organ tropism, thereby shifting early distribution^45,46^ and (iii) physicomechanical determinants of vascular wall interactions. Size, shape, and mechanical properties modulate margination and endothelial contact probability in microcirculation; particles produced during disintegration may transiently occupy size/rigidity regimes that favor wall interactions and tissue association relative to more excipient-coated colloids.^47–49^ Consistent with these mechanisms, multiple biodistribution studies of nontargeted nanoparticles report low but measurable cardiac accumulation that varies with physicochemistry and early circulation behavior, supporting a formulation-dependent component in myocardial uptake.^50,51^

This cardiac uptake in healthy animals may occur through passive targeting, where nanoparticles covalently bind to the surface of circulating cells, which are subsequently directed to the heart. Alternatively, it may result from specific binding, in which nanoparticles exhibit affinity for particular receptors on cardiac cells, leading to active targeting.^52^ Meanwhile, a brief uptake in the left lung was observed in the nanosuspension (Figure 11B).

The ritonavir nanosuspension (Figure 11B) has heterogeneous behavior among the tissues analyzed, with significantly higher values in the bladder compared to those of the right (*p* = 0.0314) and left (*p* = 0.0433) kidneys. These results indicate that, after administration, there is a rapid systemic clearance of the compound and predominant elimination via the kidneys, evidenced by high bladder uptake, suggesting efficient glomerular filtration and active urinary excretion from the system, which is corroborated in the following pharmacokinetic study. The low uptake observed in tissues such as the heart, lungs, liver, and brain suggests low tissue affinity of the formulation and transient circulation in the blood compartment, with limited penetration into organs with greater physiological barriers, such as the central nervous system, probably due to the restriction imposed by the blood–brain barrier. Finally, the low accumulation in target tissues suggests the need for adjustments in the formulation (such as surface modification, use of specific ligands or vectors) to optimize residence time and tissue targeting, depending on the therapeutic objective.

Meanwhile, ritonavir nanocrystals (Figure 11A) show a high tendency to accumulate in organs, such as the large intestine, spleen, and bladder. However, no organ showed a statistical difference when comparing the heart to the other organs, nor was there a significant difference when we made multiple comparisons between all organs. This corroborates the rapid excretion also observed in pharmacokinetic trials.

The radiopharmacokinetic assay (Figure 12), based on blood samples collected from the caudal vein of treated animals, demonstrates that both ritonavir-based systems exhibit a decrease in the blood concentration within 2 h following intraperitoneal injection. However, the nanocrystals display a transient increase at the 4 h time point before undergoing a sustained decline, ultimately mirroring the pharmacokinetic profile of the ritonavir nanosuspension.

The pharmacokinetic parameters (Table 7), including an elimination constant (*k*) of 0.1289 h^–1^ for the nanocrystal and 0.1403 h^–1^ for the nanosuspension. Additionally, a high volume of distribution (Vd) was observed, with values of 52.08 and 45.50 mL for the nanocrystal and nanosuspension, respectively. This suggests that both systems exhibit a strong ability to leave the bloodstream and distribute into extravascular compartments, corroborating the biodistribution results shown in Figure 12.^53^

**Table 7:** Pharmacokinetic Profile of Animals Treated with Ritonavir-Based Systems Radiolabeled with [^99m^Tc]

pharmacokinetics parameters	nanocrystal	nanosuspension
concentration at zero time(mg/mL)	2.589 × 10^–8^ ± 2.806 × 10^–9^	3.172 × 10^–8^ ± 5.971 × 10^–9^
elimination rate/elimination constant (k)	0.1289 ± 0.0089	0.1403 ± 0.01109
volume of distribution (mL)	52.08 ± 5.937	45.50 ± 8.320
elimination half-life(1/2)	5.470 ± 0.4378	5.024 ± 0.3533
clearance (L/h)	0.00656 ± 0.000386	0.00616 ± 0.00081

Furthermore, the elimination half-life (*t*_1/2_) was determined to be 5.4 and 5.0 h for the nanocrystal and nanosuspension, respectively. The elimination rate, or clearance (CL), was measured at 0.00656 L/h for the nanocrystal and 0.00616 L/h for the nanosuspension.

##### 3.5.2.1 In Vitro–In Vivo Relationship

The dissolution studies in POE10LE (0.06 and 0.04 M) show that the nanosuspension dissolves essentially instantaneously, whereas spray-dried nanoaggregates require an initial disintegration step before complete dissolution; DE0–90 ranking is NSP > NCR (spray-dried) > API (Table 4; Figures 9, 10). The in vivo, after intraperitoneal dosing, both lead formulations exhibit similar overall blood profiles, with a transient increase at 4 h observed for the nanocrystal group before convergence (Figure 12).

Given the intraperitoneal route and the colloidal nature of the systems, systemic exposure reflects not only dissolution but also aggregate disintegration, peritoneal clearance, mononuclear phagocyte system uptake, and hepatobiliary recirculation. Under these conditions, a Level A IVIVC (in vitro-in vivo correlation) (point-to-point) is not expected. Instead, the data support a qualitative IVIVR: the in vitro observation that nanoaggregates must disintegrate before fully dissolving is consistent with the 4 h in vivo peak, whereas the rapidly dissolving nanosuspension shows a monotonic decline without a secondary rise.

#### 3.5.3 Biochemical Analysis

The results of the biochemical analyses are summarized in Table 8. The evaluated parameters include alanine aminotransferase (ALT), aspartate aminotransferase (AST), gamma-glutamyl transferase (GGT), creatinine (CRE), cholesterol (CHOL), lipase (LPS), amylase (AMS), and glucose (GLU).^54–56^

**Table 8:** Biochemical Analysis of Healthy Animals Treated with Ritonavir Nanocrystal and Nanosuspension

nanocrystal	ALT (U/L)	AST (U/L)	GGT (U/L)	CRE (mg/dL)	CHOL	LPS (mg/dL)	AMS (U/L)	GLU (mg/dL)
average	62.4	25.43	12.5	0	42.93	627.8	519.5	7.533
SEM	15.02	24.68	3.066	0	21.45	230.5	119	2.619
nanosuspension	ALT (U/L)	AST (U/L)	GGT (U/L)	CRE (mg/dL)	CHOL	LPS (mg/dL)	AMS (U/L)	GLU (mg/dL)
average	14.13	0.7	25.5	0	52	478.6	107	3.8
SEM	1.713	0.1528	7.062	0	21.62	147.8	38.86	1.185
reference	ALT (U/L)	AST (U/L)	GGT (U/L)	CRE (mg/dL)	CHOL	LPS (mg/dL)	AMS (U/L)	GLU (mg/dL)
average	46.15	121.60	3.76	0.57	90.20	-	72.0	71.3
SEM	5.62	35.93	0.11	0.06	6.13	-	1.5	1.0

The liver is a vital organ that plays a central role in metabolism, digestion, detoxification, and excretion of endogenous and exogenous substances. Alanine aminotransferase (ALT) is a key enzyme predominantly synthesized in hepatocytes, though it is also present in other tissues, including the kidneys, heart, and skeletal muscle. Persistently elevated ALT levels may serve as a biomarker of hepatocellular injury, reflecting underlying liver dysfunction or disease.^57^ Similarly, aspartate aminotransferase (AST) is an enzyme localized in the cytoplasm and mitochondria of hepatocytes as well as in skeletal muscle, cardiac muscle, kidneys, and other tissues. Hepatocellular injury or damage to other AST-rich tissues can lead to the release of this enzyme into the bloodstream, resulting in elevated serum AST levels. Therefore, an increase in AST may serve as a biochemical marker of tissue injury, with its specificity depending on the context and concurrent elevation of other biomarkers, such as ALT.^58^ Our results indicate that the administration of ritonavir nanocarriers did not induce significant alterations in ALT or AST levels. This suggests that the nanocarriers do not elicit hepatocellular injury or compromise liver function under the conditions evaluated.

On the other hand, we evaluated γ-glutamyltransferase (GGT), an enzyme crucial for glutathione metabolism, predominantly located in the membranes of hepatocytes and the epithelium of bile ducts. Although GGT is also present in various organs, including the kidneys, spleen, heart, pancreas, and brain, its elevated serum levels are frequently correlated to increased alkaline phosphatase (ALP) activity. Consequently, GGT serves as a valuable biomarker in liver function assessment, particularly in conditions affecting biliary tract integrity and hepatic oxidative stress.^59^ In this regard, we observed a significant increase in GGT levels 24 h postinjection, specifically in the nanosuspension-treated group. This elevation may indicate hepatic stress or potential hepatobiliary dysfunction, suggesting that the nanosuspension formulation could exert an effect on liver physiology. Further investigation is warranted to elucidate the underlying mechanisms and determine whether this effect is transient or indicative of sustained hepatic injury.^60^

Next, we evaluated renal function by measuring plasma creatinine (CRE) levels in the treated animals. CRE is a metabolic byproduct of muscle catabolism that is primarily filtered by the glomeruli and excreted by the kidneys. Under normal physiological conditions, its serum concentration remains relatively stable as it is efficiently eliminated by the renal system. Therefore, alterations in CRE levels may serve as an indicator of impaired renal function or altered glomerular filtration.^61^ In the in vivo assay, creatinine was not detected (value = 0), suggesting the absence of renal impairment despite the active renal filtration of both nanocarriers, as evidenced by the biodistribution data. This finding indicates that nanocarriers do not induce detectable nephrotoxicity under the conditions tested, reinforcing their potential biocompatibility in terms of renal function.

To gain preliminary insights into lipid metabolism, we analyzed blood cholesterol levels, a key biomarker associated with lipid homeostasis and cardiovascular risk. Cholesterol is essential for cellular function, hormone synthesis, and membrane integrity; however, dysregulation in its levels is strongly linked to the development and progression of cardiovascular diseases.^62^ Based on this parameter, we observed that neither ritonavir-based nanocarrier-induced alterations in blood cholesterol levels, suggesting no adverse effects on lipid metabolism or cardiovascular health in the treated animals. This finding is further supported by the stability of ALT levels and biodistribution data, reinforcing the overall biocompatibility of the formulations under the conditions evaluated.

Amylase is a key digestive enzyme primarily produced by the pancreas and salivary glands. Its primary function is to catalyze the hydrolysis of glycosidic bonds in starch, facilitating the breakdown of complex polysaccharides into simpler carbohydrates, such as maltose and glucose. This enzymatic activity is essential for carbohydrate digestion and nutrient absorption in the gastrointestinal tract.^63^ Our data reveal a significant elevation in amylase levels compared to reference values, with an even more pronounced increase observed in the nanocrystal-treated group. This finding suggests a potential impact of the nanocarrier formulations on the pancreatic function or gastrointestinal enzyme regulation. Further investigation is warranted to determine whether this increase reflects transient physiological adaptation or is an indication of pancreatic stress.

To assess whether ritonavir nanocarriers influence simple carbohydrate metabolism, we measured blood glucose levels. Glucose is a fundamental energy substrate and a precursor for the biosynthesis of various essential biomolecules, including glycogen, ribose, deoxyribose, galactose, glycolipids, glycoproteins, and proteoglycans.^64^ Our results demonstrated a significant alteration in glucose levels, aligning with the observed changes in amylase activity. This correlation suggests a potential interaction between the nanocarriers and metabolic pathways involved in carbohydrate digestion and utilization, warranting further investigation into the underlying mechanisms.

Certain nanostructures, such as nanoparticles, have been shown to influence glucose metabolism.^65^ For instance, Chen and collaborators (2014) demonstrated that polyvinylpyrrolidone-coated silver nanoparticles (PVP-AgNPs) can reprogram cellular energy metabolism by altering the expression of key metabolic genes.^66^ These nanoparticles suppress peroxisome proliferator-activated receptor α-coactivator 1-alpha (PGC-1α), leading to disruptions in the Krebs cycle and lipid metabolism. Additionally, they downregulate pyruvate dehydrogenase (PDH), thereby inhibiting the conversion of pyruvate to acetyl-CoA and impairing mitochondrial oxidative phosphorylation.

As a compensatory mechanism, cells upregulate phosphofructokinase-2/fructose-2,6-bisphosphatase 3 (PFKFB3), enhancing glycolysis to counteract the reduced level of ATP production and sustain cellular energy demands. This metabolic shift results in increased pyruvate and lactate accumulation while simultaneously decreasing ATP synthesis.^66^ Given that lactate and pyruvate can serve as substrates for gluconeogenesis, this pathway may offer a plausible explanation for the glucose level alterations observed in our study. Further investigation is needed to determine whether ritonavir nanocarriers induce similar metabolic adaptations.

The formulation-dependent elevation in GGT observed predominantly with the nanosuspension suggests a mild, formulation-triggered hepatobiliary stress at 24 h postdose, consistent with GGT’s role as a sensitive indicator of cholestatic perturbation and biliary epithelial stress, which can be observed in the use of ritonavir.^67,68^ While ALT/AST remained unchanged—arguing against overt hepatocellular injury—the selective rise in GGT points to differences in how the two drug products interface with the hepatobiliary axis, potentially via differential uptake by Kupffer cells or biliary epithelium and/or distinct patterns of biliary excretion of colloidal material.

By contrast, the stronger increase in amylase with the nanocrystal formulation carries pancreatic significance. Although nonspecific elevations of amylase can occur,^69^ the directionally concordant perturbation in circulating glucose implies a formulation-linked impact on carbohydrate handling. Together, these data are compatible with a transient, formulation-mediated modulation of digestive enzyme release and systemic glucose homeostasis at 24 h, aligning with prior evidence that nanostructures can reprogram cellular energy metabolism toward glycolysis.^66,70,71^

Mechanistically, we hypothesize that differences in particle architecture and surface chemistry between the nanocrystal and nanosuspension underlie these divergent metabolic fingerprints. The nanocrystal—comprised almost entirely of API and stabilized by low levels of polymeric steric agents—presents high interfacial curvature and dense API surface, which may favor interaction with exocrine pancreatic tissues or gut-associated compartments following intraperitoneal dosing, amplifying amylase release, and secondarily altering glucose. In contrast, the nanosuspension, with a higher fraction of freely dispersed submicron particulates and excipient exposure at the interface, may traffic more avidly through the hepatobiliary route, leading to selective GGT elevations without parallel ALT/AST changes. This interpretation is consistent with the documented liver/kidney uptake at 24 h, the absence of creatinine changes, and the lack of ALT/AST perturbation.

The contributory role of specific excipients is also plausible. Polymeric steric stabilizers (e.g., HPC grades used during milling) can modulate protein adsorption and opsonization, altering hepatic versus pancreatic tissue exposure;^72,73^ conversely, residual surfactants used elsewhere in the workflow (e.g., POE10LE in dissolution media) highlight how surfactant chemistry can influence biological interfaces, although direct carryover into the in vivo study is not indicated here.^74^ We therefore posit that (i) particle surface composition (API-rich vs excipient-coated), (ii) hydrodynamic size/aggregation state, and (iii) steric layer identity jointly shape biliary versus pancreatic engagement, yielding the observed GGT-dominant signal in the nanosuspension and the amylase/glucose-dominant signal in the nanocrystal group. These mechanistic hypotheses are consistent with literature showing nanobio interface-driven shifts toward glycolysis and altered intermediary metabolism.

## 4 Conclusions

In this study, RTV nanocrystals were successfully developed, demonstrating significant improvements in dissolution and stability. These nanocrystals represent a promising platform for future pharmaceutical applications.

The optimized formulation produced nanoscale particles with stability against agglomeration. The use of the Ultra-Turrax Tube Drive disperser, in combination with milling beads, proved to be an efficient and practical method for initial size reduction, facilitating scale-up. Beads of 500 and 200 μm were identified as the most suitable for RTV milling, while combining different bead sizes did not yield substantial improvements, although this approach may benefit the processing of other APIs. Spray drying was effective in producing nanoaggregated microparticles while preserving the nanocrystal structure.

For characterization, the combined use of laser diffraction and dynamic light scattering allowed for accurate particle size monitoring. Additional analyses confirmed that RTV remained highly stable, retaining its crystalline form (form II) throughout processing.

Dissolution studies showed that nanosuspensions dissolved immediately, whereas nanoaggregates required an initial disintegration step. The dissolution medium significantly influenced discrimination between formulations, and this finding is particularly relevant from a health surveillance perspective, as the pharmacopeial dissolution media developed for conventional drugs (0.06 M POE10LE) required medium concentrations close to saturation (0.04 M POE10LE) to differentiate between the dispersed API and dried nanocrystals. This need underscores the importance of revisiting and adapting official methodologies to accommodate new nanoformulations, thereby ensuring the quality and safety of the final products.

Radiolabeling showed over 90% efficiency, indicating good stability and durability. Biodistribution studies revealed uptake in the kidneys, liver, and intestines, with notable differences in cardiac absorption between nanocrystals and nanosuspension. Pharmacokinetic analysis suggested similar distribution profiles, with a rapid decline in blood concentration within 2 h and a slight increase in nanocrystal levels at 4 h, followed by gradual elimination. Biochemical analyses indicated no significant changes in ALT and AST levels; however, the nanosuspension led to a marked increase in GGT, suggesting a potential hepatic impact. Both nanosystems affected carbohydrate metabolism, as evidenced by increased amylase and glucose levels—more pronounced in the nanocrystal group.

Overall, these findings underscore the potential of RTV nanocrystals for pharmaceutical use, while highlighting the need for further studies. Future research should focus on their long-term stability, in vivo efficacy, and metabolic effects to ensure safety and maximize therapeutic benefits.

## Supplementary Material



## Notes

Complete time-course results of the milling processes, with particle size and process monitoring data collected at 30 min intervals; influence of the Ultra-Turrax on the milling of presuspensions, both without and in combination with 500 μm beads; and SEM images showing the morphology of the nanosuspension prior to spray-drying (PDF)
