# Supplementary material for: Optimized Milling Approaches for Scalable Production of Ritonavir Nanocrystals: from Process Design to Bioperformance Evaluation
Source: ACS Omega. 2026 Jan 27;11(5):7908–25. doi: 10.1021/acsomega.5c09971 (PMC12902989; doi:10.1021/acsomega.5c09971)
Supplement: Supplementary file 1 [file ao5c09971_si_001.pdf]

Supporting Information

Optimized milling approaches for scalable production of ritonavir nanocrystals: from process design to bioperformance evaluation

Marcelo Henrique da Cunha Chaves[a,b,*] Francisco Alexandrino Júnior[a]; Michelle Alvares Sarcinelli[a]; Natalia Cristina Gomes-da-Silva[c]; Ralph Santos-Oliveira[c,d]; Fabio Coelho Amendoeira [b]; Helvécio Vinícius Antunes Rocha[a,b]

[a] Laboratory of Micro and Nanotechnology, Oswaldo Cruz Foundation/Fiocruz, Rio de Janeiro, RJ, Brazil.

[b] Postgraduate Program in Health Surveillance, National Institute for Quality Control in Health, Oswaldo Cruz Foundation/Fiocruz, Rio de Janeiro, RJ, Brazil.

[c] Brazilian Nuclear Energy Commission, Nuclear Engineering Institute, Laboratory of Nanoradiopharmacy and Synthesis of New Radiopharmaceuticals, Rio de Janeiro-RJ, 21941906, Brazil.

[d] State University of Rio de Janeiro, Laboratory of Nanoradiopharmacy and Strategic Biomaterials, Rio de Janeiro-RJ, 220000, Brazil.

*Corresponding author. E-mail address: marcelo.chaves@fiocruz.br.

Supporting Information Table 1. Evaluation of different bead sizes (500, 200, and 100 μm) in the milling of pre-suspensions.

Sample	LD				DLS		
	D10 (μm)	D50 (μm)	D90 (μm)	span	Z-ave (nm)	PDI	ZP (mV)
500 μm Beads							
Unmilled sample	5.170	15.000	1310.000	86.567	-	-	-
30 min milling	0.592	5.310	431.000	81.078	-	-	-
60 min milling	0.355	1.460	5.390	3.445	-	-	-
90 min milling	0.273	0.572	1.780	2.630	-	-	-
120 min milling	0.248	0.517	1.340	2.117	-	-	-
150 min milling	0.246	0.504	1.220	1.935	509.3	0.223	-38.9
180 min milling	0.229	0.473	1.160	1.971	474.7	0.263	-38.5
210 min milling	0.227	0.464	1.110	1.895	436.6	0.237	-42.6
240 min milling	0.210	0.429	0.916	1.644	429.1	0.221	-42.2
270 min milling	0.204	0.418	0.870	1.593	402.8	0.197	-40.2
300 min milling	0.201	0.410	0.843	1.568	396.7	0.166	-36.8
Stability (7 days)	0.203	0.410	0.821	1.510	381.9	0.225	-34.3
200 μm Beads							
Unmilled sample	4.710	11.800	46.800	3.575	-	-	-
30 min milling	0.621	2.600	7.331	2.799	-	-	-
60 min milling	0.234	0.488	1.170	1.919	-	-	-
90 min milling	0.213	0.432	0.904	1.598	-	-	-
120 min milling	0.188	0.387	0.776	1.517	-	-	-
150 min milling	0.178	0.366	0.713	1.463	353.8	0.205	-48.3
180 min milling	0.168	0.345	0.664	1.436	325.8	0.108	-31.4
210 min milling	0.162	0.331	0.631	1.417	317.0	0.181	-37.4
240 min milling	0.155	0.321	0.611	1.421	319.0	0.164	-37.8
270 min milling	0.151	0.312	0.593	1.420	300.9	0.161	-40.2
300 min milling	0.152	0.310	0.585	1.397	300.7	0.170	-30.0
Stability (7 days)	0.151	0.303	0.559	1.344	293.7	0.146	-37.8
100 μm Beads							
Unmilled sample	5.050	12.500	49.300	3.551	-	-	-
30 min milling	2.580	7.150	24.400	3.051	-	-	-
60 min milling	1.440	5.610	18.700	3.085	-	-	-
90 min milling	0.746	4.210	13.900	3.114	-	-	-
120 min milling	0.557	3.290	11.300	3.269	-	-	-
150 min milling	0.456	2.590	10.500	3.886	-	-	-
180 min milling	0.357	1.780	8.930	4.805	-	-	-
210 min milling	0.309	1.290	7.730	5.752	-	-	-
240 min milling	0.264	0.986	6.680	6.503	-	-	-
270 min milling	0.266	0.565	1.580	2.321	572.4	0.320	-39.0
300 min milling	0.267	0.559	1.390	2.013	533.6	0.253	-40.8
Stability (7 days)	0.245	0.490	1.030	1.599	472.0	0.256	-40.0

Supporting Information Table 2. Influence of TURRAX (11,000 RPM) on the milling of pre-suspensions.

Sample	LD				DLS		
	D10 (µm)	D50 (µm)	D90 (µm)	span	Z-Ave (nm)	PDI	ZP (mV)
Ultra-Turrax T 25							
Unmilled sample	3.990	11.200	41.400	3.328	-	-	-
3 min milling	3.680	10.600	39.100	3.339	-	-	-
7 min milling	3.700	10.800	39.900	3.342	-	-	-
15 min milling	3.550	10.400	37.500	3.273	-	-	-

Supporting Information Table 3. Combined milling evaluation of TURRAX (18,000 RPM) and 500 µm beads in the milling of pre-suspensions.

Sample	LD				DLS		
	D10 (µm)	D50 (µm)	D90 (µm)	span	Z-Ave (nm)	PDI	ZP (mV)
Unmilled sample	5.090	12.600	50.100	3.579	-	-	-
3 min milling with TURRAX	4.350	10.900	38.800	3.163	-	-	-
7 min milling with TURRAX	4.190	10.600	37.200	3.109	-	-	-
30 min milling with beads	0.691	3.440	18.900	5.282	-	-	-
60 min milling with beads	0.466	1.500	6.950	4.317	-	-	-
90 min milling with beads	0.368	0.953	5.350	5.229	-	-	-
120 min milling with beads	0.277	0.570	1.370	1.908	-	-	-
150 min milling with beads	0.254	0.516	1.160	1.758	-	-	-
180 min milling with beads	0.240	0.488	1.070	1.699	438.3	0.242	-40.4
210 min milling with beads	0.229	0.472	1.060	1.764	434.1	0.244	-37.1
240 min milling with beads	0.229	0.463	0.987	1.638	407.9	0.203	-37.3
270 min milling with beads	0.219	0.441	0.900	1.543	393.7	0.201	-38.0
300 min milling with beads	0.212	0.427	0.862	1.525	378.3	0.218	-37.7
Stability (7 days)	0.202	0.407	0.793	1.452	360.8	0.181	-37.7

Supporting Information Table 4. Combination of bead sizes in the milling process of pre-suspensions.

Sample	LD				DLS		
	D10 (µm)	D50 (µm)	D90 (µm)	span	Z-Ave (nm)	PDI	ZP (mV)
Bead combination: 500 µm + 200 µm							
Unmilled sample	4.980	11.700	34.400	2.504	-	-	-
240 min milling with 500 µm beads	0.216	0.437	0.895	1.551	397.5	0.220	-33.4
30 min milling with 200 µm beads	0.196	0.394	0.760	1.433	347.2	0.193	-34.2

60 min milling with 200 µm beads	0.184	0.372	0.713	1.426	332.1	0.208	-36.2
90 min milling with 200 µm beads	0.176	0.357	0.678	1.407	322.6	0.201	-35.8
120 min milling with 200 µm beads	0.173	0.347	0.651	1.377	314.7	0.186	-34.7
150 min milling with 200 µm beads	0.172	0.342	0.632	1.346	315.5	0.155	-37.9
180 min milling with 200 µm beads	0.167	0.333	0.616	1.349	303.7	0.181	-34.6
210 min milling with 200 µm beads	0.164	0.328	0.612	1.367	302.2	0.167	-35.8
240 min milling with 200 µm beads	0.168	0.334	0.626	1.372	302.5	0.156	-36.3
Stability (7 days)	0.165	0.329	0.610	1.355	305.4	0.156	-33.7
Bead combination: 500 µm + 100 µm							
Unmilled sample	5.120	12.400	47.700	3.436	-	-	-
240 min milling with 500 µm beads	0.219	0.445	0.935	1.612	419.3	0.173	-37.3
30 min milling with 100 µm beads	0.215	0.434	0.899	1.577	379.6	0.182	-37.5
60 min milling with 100 µm beads	0.194	0.392	0.763	1.452	379.4	0.186	-37.8
90 min milling with 100 µm beads	0.192	0.389	0.759	1.456	337.0	0.189	-38.3
120 min milling with 100 µm beads	0.187	0.378	0.723	1.419	326.2	0.187	-34.9
150 min milling with 100 µm beads	0.185	0.375	0.723	1.437	322.6	0.188	-34.4
180 min milling with 100 µm beads	0.181	0.366	0.709	1.443	320.2	0.154	-32.8
210 min milling with 100 µm beads	0.178	0.358	0.678	1.397	317.8	0.130	-36.8
240 min milling with 100 µm beads	0.170	0.342	0.641	1.378	304.1	0.134	-35.2
Stability (7 days)	0.155	0.312	0.574	1.342	293.8	0.129	-39.2

Supporting Information Table 5. Results of the evaluation of the use of steric stabilizers in the milling of pre-suspensions.

**Sample**	**LD**				**DLS**		
	**D10 (µm)**	**D50 (µm)**	**D90 (µm)**	**span**	**Z-Ave (nm)**	**PDI**	**ZP (mV)**
**HPC LF**							

Unmilled sample	4.690	11.700	41.800	3.177	-	-	-
30 min milling	0.307	0.732	2.940	3.592	-	-	-
60 min milling	0.283	0.636	2.080	2.828	-	-	-
90 min milling	0.267	0.579	1.630	2.348	-	-	-
120 min milling	0.252	0.544	1.470	2.248	635.5	0.275	-30.1
150 min milling	0.252	0.535	1.320	2.001	591.8	0.264	-32.9
180 min milling	0.235	0.494	1.260	2.071	553.5	0.277	-34.6
210 min milling	0.222	0.467	1.070	1.819	509.7	0.224	-34.5
240 min milling	0.221	0.455	0.992	1.693	512.4	0.259	-33.1
270 min milling	0.218	0.447	0.971	1.686	488.4	0.253	-29.6
300 min milling	0.205	0.428	0.929	1.689	472.5	0.229	-33.4
Stability (7days)	0.216	0.450	1.010	1.762	548.3	0.279	-32.8
**HPC SSL**							
Unmilled sample	4.760	11.800	42.700	3.218	-	-	-
30 min milling	0.298	0.683	2.390	3.060	-	-	-
60 min milling	0.275	0.594	1.680	2.370	-	-	-
90 min milling	0.251	0.533	1.350	2.066	-	-	-
120 min milling	0.239	0.501	1.190	1.890	469.1	0.348	-38.1
150 min milling	0.225	0.468	1.050	1.772	443.6	0.257	-39.9
180 min milling	0.220	0.452	0.965	1.650	408.8	0.228	-39.4
210 min milling	0.212	0.434	0.905	1.596	375.1	0.231	-32.7
240 min milling	0.201	0.417	0.863	1.589	375.2	0.234	-38.8
270 min milling	0.198	0.406	0.827	1.549	364.9	0.242	-39.1
300 min milling	0.196	0.399	0.794	1.500	351.3	0.205	-38.0
Stability (7days)	0.191	0.394	0.802	1.551	358.9	0.202	-34.7
**HPMC 603**							
Unmilled sample	4.780	11.700	39.400	2.953	-	-	-
30 min milling	0.477	2.970	7.690	2.430	-	-	-
60 min milling	0.237	1.160	4.910	4.041	-	-	-
90 min milling	0.142	0.421	1.370	2.929	-	-	-
120 min milling	0.140	0.398	1.170	2.589	531.1	0.304	-29.8
150 min milling	0.151	0.400	1.090	2.357	480.0	0.271	-33.6
180 min milling	0.105	0.320	0.907	2.506	443.5	0.311	-32.4
210 min milling	0.135	0.360	0.940	2.233	427.1	0.229	-36.6
240 min milling	0.124	0.338	0.893	2.274	403.1	0.222	-35.3
270 min milling	0.094	0.282	0.753	2.341	390.1	0.223	-35.1
300 min milling	0.090	0.272	0.733	2.365	377.0	0.239	-34.6
Stability (7days)	0.243	0.532	11.100	20.316	380.4	0.194	-32.2
**POL188**							
Unmilled sample	4.710	11.700	40.500	3.062	-	-	-
30 min milling	1.420	4.310	12.200	2.495	-	-	-
60 min milling	0.302	0.649	2.330	3.122	-	-	-
90 min milling	0.284	0.610	2.390	3.453	-	-	-

120 min milling	0.261	0.558	1.650	2.487	632.8	0.328	-37.0
150 min milling	0.265	0.551	1.720	2.638	600.5	0.341	-35.8
180 min milling	0.258	0.532	1.580	2.477	551.6	0.258	-33.6
210 min milling	0.253	0.518	1.550	2.507	545.4	0.343	-32.8
240 min milling	0.242	0.493	1.250	2.045	512.3	0.314	-33.7
270 min milling	0.240	0.496	1.730	3.007	523.4	0.307	-34.4
300 min milling	0.235	0.483	1.590	2.815	494.3	0.265	-33.2
Stability (7days)	0.218	0.464	1.570	2.911	484.1	0.251	-32.5
**POL407**							
Unmilled sample	3.680	10.800	30.600	2.501	-	-	-
30 min milling	1.580	4.240	11.000	2.218	-	-	-
60 min milling	0.303	0.678	3.000	3.979	-	-	-
90 min milling	0.290	0.632	2.460	3.425	-	-	-
120 min milling	0.271	0.582	1.730	2.503	656.2	0.355	-36.6
150 min milling	0.271	0.563	1.720	2.582	621.5	0.339	-36.2
180 min milling	0.261	0.542	1.710	2.673	598.4	0.328	-32.9
210 min milling	0.242	0.506	1.320	2.127	576.2	0.289	-34.3
240 min milling	0.246	0.510	1.810	3.060	552.8	0.281	-32.9
270 min milling	0.242	0.499	1.680	2.881	543.7	0.262	-31.9
300 min milling	0.234	0.482	1.470	2.555	519.5	0.273	-32.1
Stability (7days)	0.223	0.480	1.840	3.369	530.5	0.266	-32.4
**SSG**							
Unmilled sample	10.500	114.000	224.000	1.876	-	-	-
30 min milling	7.620	111.000	1880.000	16.814	-	-	-
60 min milling	5.170	135.000	620.000	4.546	-	-	-
**CCS**							
Unmilled sample	6.040	19.300	102.000	4.964	-	-	-
30 min milling	2.720	42.000	192.000	4.509	-	-	-
60 min milling	2.720	25.200	1930.000	76.424	-	-	-

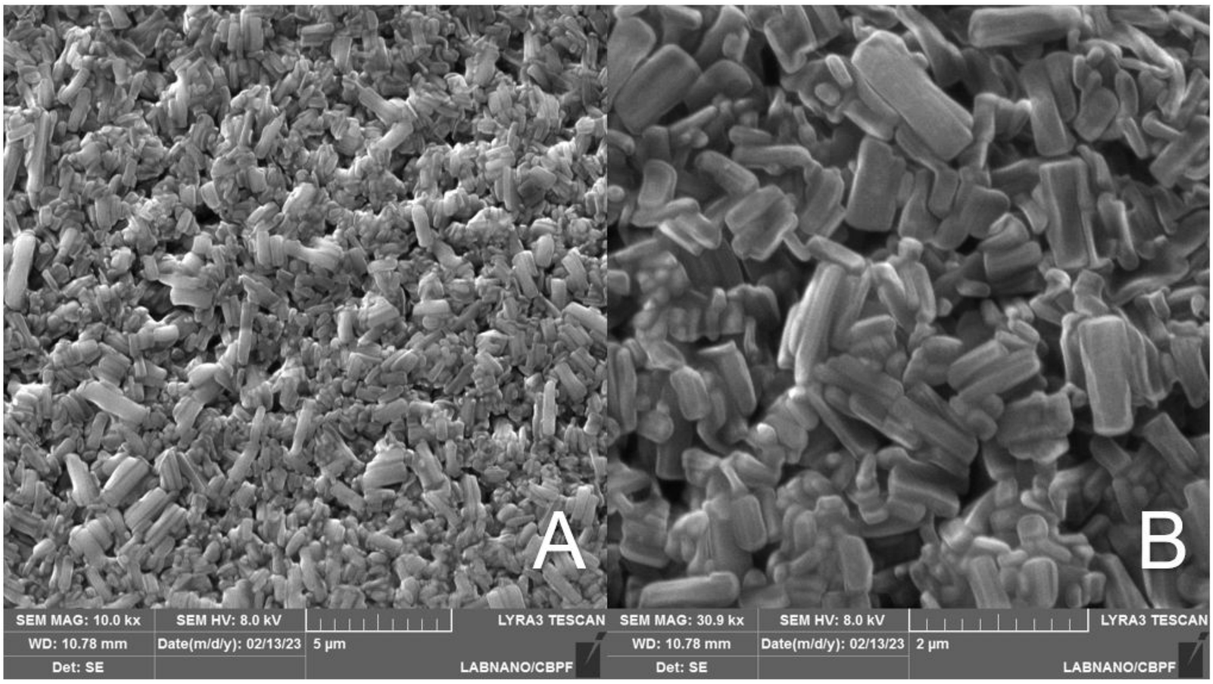

Supporting Information Figure 1. SEM of RTV nanossuspension. A: magnification x10000; B: magnification x30900
